# Functional neuroanatomy of musical object processing in Alzheimer’s disease and frontotemporal dementia

**DOI:** 10.1093/braincomms/fcag161

**Published:** 2026-05-05

**Authors:** Lucy B Core, Sophie A Froud, Stephen Wastling, Jessica Jiang, Benjamin A Levett, Laura Mancini, Barbara Dymerska, Chris J D Hardy, Peter Zeidman, Jason D Warren

**Affiliations:** Brain Behaviour Group, Dementia Research Centre, Queen Square Institute of Neurology, University College London, London WC1N 3AR, United Kingdom; Brain Behaviour Group, Dementia Research Centre, Queen Square Institute of Neurology, University College London, London WC1N 3AR, United Kingdom; National Hospital for Neurology and Neurosurgery, University College London Hospitals National Health Service Foundation Trust, London WC1N 3BG, United Kingdom; Department of Translational Neuroscience and Stroke, Queen Square Institute of Neurology, University College London, London WC1N 3AR, United Kingdom; Brain Behaviour Group, Dementia Research Centre, Queen Square Institute of Neurology, University College London, London WC1N 3AR, United Kingdom; Brain Behaviour Group, Dementia Research Centre, Queen Square Institute of Neurology, University College London, London WC1N 3AR, United Kingdom; National Hospital for Neurology and Neurosurgery, University College London Hospitals National Health Service Foundation Trust, London WC1N 3BG, United Kingdom; Department of Imaging Neuroscience, Queen Square Institute of Neurology, University College London, London WC1N 3AR, United Kingdom; Brain Behaviour Group, Dementia Research Centre, Queen Square Institute of Neurology, University College London, London WC1N 3AR, United Kingdom; Department of Imaging Neuroscience, Queen Square Institute of Neurology, University College London, London WC1N 3AR, United Kingdom; Brain Behaviour Group, Dementia Research Centre, Queen Square Institute of Neurology, University College London, London WC1N 3AR, United Kingdom

**Keywords:** music, Alzheimer’s disease, frontotemporal dementia, fMRI, auditory object

## Abstract

Music, besides its emotional and social resonance, models a complex sensory environment exemplifying auditory objects corresponding to sources (musical instruments) and information streams (melodies). These musical dimensions can be variably preserved or blighted by neurodegenerative disease. Music is, therefore, an attractive way to investigate the neural mechanisms of sensory object processing in these diseases. Here, we assessed the functional neuroanatomy underlying the perceptual, semantic, and apperceptive processing of musical objects in Alzheimer’s disease and temporal variant frontotemporal dementia. We studied 35 patients (20 Alzheimer’s disease, 15 temporal variant frontotemporal dementia; 14 females; mean [standard deviation] age 70.3 [8.4] years) in relation to 25 cognitively healthy volunteers (16 females; age 69.5 [6.8] years). In a functional MRI experiment with sparse image acquisition to minimise the impact of scanner noise, participants passively listened to monophonic melodies. We varied timbre (same/change), timbre familiarity (natural/artificial instruments), melody familiarity (familiar/novel), and apperception (melodies with interpolated timbre changes); these manipulations allowed us to assess the functional neuroanatomical correlates of musical feature perception, melody familiarity, constancy, novelty, and instrument familiarity. Behavioural correlates were assessed in post-scan tasks and disease-related atrophy patterns using voxel-based morphometry of participants’ structural scans. All contrasts were assessed at *P* < 0.05, corrected for multiple voxel-wise comparisons within pre-specified anatomical regions of interest. For timbre change perception, all participant groups demonstrated comparable temporo-parietal cortical activation anchored in planum temporale. For melodies, processing of semantic familiarity in all participant groups engaged a common network including supplementary motor area and inferior frontal gyrus, with reduced supplementary motor area activation in the Alzheimer’s disease group compared with other groups, while melody novelty comparably engaged postero-medial cortical circuitry across groups. Apperceptive coding of melody constancy was associated with activation of the posterior superior temporal cortex in the healthy volunteers, but greater activation of the temporal polar cortex in the temporal variant frontotemporal dementia group than in healthy volunteers. For instrument familiarity, the temporal variant frontotemporal dementia group showed reduced activation of the temporal polar cortex, but increased activation of the anterior insula compared to healthy volunteers. Brain activation profiles were not influenced by behavioural performance on post-scan tasks and did not coincide with regional atrophy. Our findings delineate complex, differentiated functional neuroanatomical profiles of musical object processing in Alzheimer’s disease and frontotemporal dementia, with implications for our understanding of the neural mechanisms that decode complex sensory environments and the design and evaluation of interventions in these diseases.

## Teaser

Core *et al*. employed a functional MRI paradigm investigating the perceptual, semantic and apperceptive components of musical object processing in Alzheimer’s disease and frontotemporal dementia. They found differentiated syndromic neuroanatomical profiles of musical instrument and melody cognition, highlighting how neurodegeneration alters brain mechanisms supporting the perception of complex sensory environments.

## Graphical Abstract

**Graphical Abstract:**
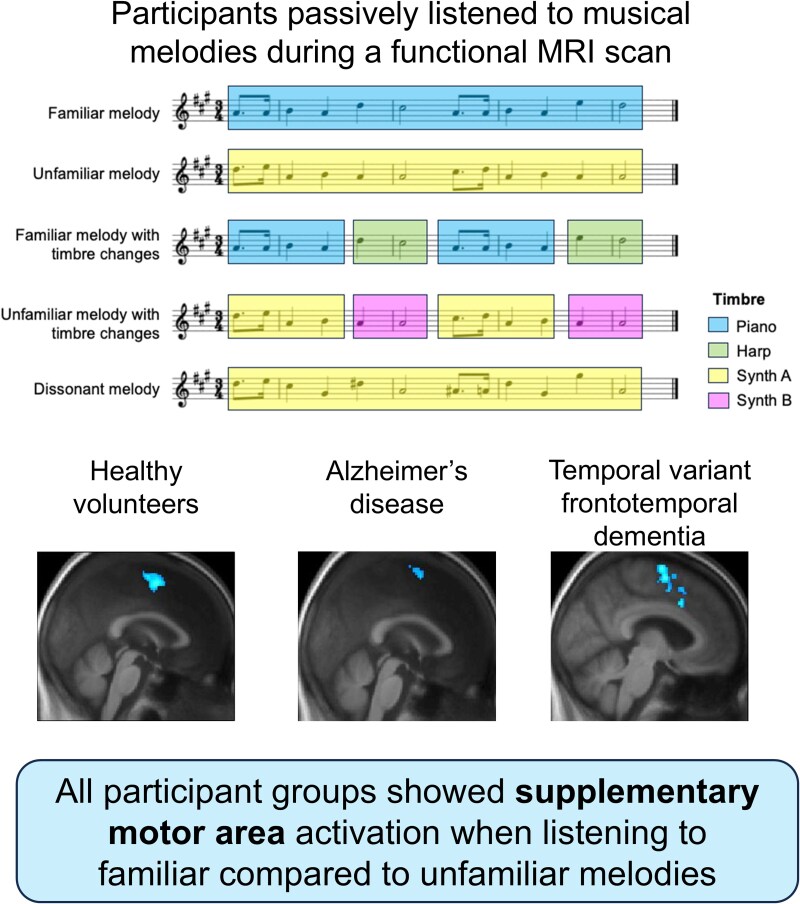

## Introduction

Transforming the chaotic sensory world into meaningful objects is a fundamental task of the brain. Sensory object perception is a multi-componential, computationally intensive process that depends on integrative activity in distributed brain circuitry. As such, it is vulnerable in neurodegenerative diseases such as Alzheimer’s disease (AD) and frontotemporal dementias (FTD). People with these diseases frequently develop difficulties navigating, perceiving and understanding complex everyday sensory environments, from early stages of the illness.^1-6^ Impaired binding of perceptual features into stable representations of whole sensory objects is a neuropsychological hallmark of AD.^7-10^ In the FTD spectrum, the semantic variant of primary progressive aphasia (svPPA) is the archetypal disorder of semantic memory, associated with impaired comprehension of sensory object concepts.^11,12^ These deficits reflect the neural networks principally targeted by AD and svPPA: in AD, the temporo-parietal default mode network^13-15^ and in svPPA, the semantic appraisal network anchored in the anterior temporal lobe.^16,17^ Sensory object processing might therefore illuminate the core pathophysiology of these diseases, with implications for diagnosis and designing interventions. However, the neural mechanisms that mediate sensory object perception deficits in major dementias remain poorly characterized.

Music is an attractive model paradigm for addressing the neural bases of sensory object processing in AD and FTD. Though less familiar than visual objects, auditory objects—broadly defined as collections of acoustic data bound in common perceptual representations and disambiguated from the auditory scene^18,19^—are of profound importance in daily life. Music exemplifies auditory objects corresponding both to sound sources (the particular sound quality of a musical instrument conveyed by timbre, such as the characteristic sound of a piano)^20,21^ and source-invariant sound patterns or information streams (the pitch sequences that comprise melodies, such as the specific sequence of notes in ‘*Happy Birthday’*).^22-24^ Source (instrument) and source-invariant (melody) auditory object characteristics normally interact to constitute a coherent percept of a musical piece.^25^ As a special class of auditory objects, music approaches speech phonemes in that it has ‘rules’ that govern how musical objects are constituted, transformed and sequenced, unlike many other classes of nonverbal sounds. These rules are internalized by listeners through exposure to music, even without musical training.^26^ Music also has ecological relevance and universality; its emotional and reward value may be even more potent, and its social and communicative functions are distinct from language.^27,28^

From a neuropsychological perspective, musical (like other auditory) objects engage distinct, hierarchically organized (but reciprocal and dissociable) neural processing stages.^18,22^ These stages include the encoding of auditory properties (feature detection and perception), representation of sets of perceptual features corresponding to whole objects (apperception), and object recognition (semantic association). Importantly, musical melodies can be altered in various ways (for example, transposed in pitch or key, or distributed among different instruments) while retaining their identity. Thus, melodies may serve as a model for auditory ‘object constancy’, an auditory analogue of visual apperceptive processing, whereby the same object viewed under different conditions is identified by matching to a stored, stable neural template representation.^11,18,29-32^ Decoding emotional attributes of musical objects is likely to depend on neural mechanisms that are at least partly music-specific and in parallel to other processing stages. Dissonance may serve as a prototypical driver of music-specific emotional states,^33-38^ as music containing notes that do not fit the prevailing key tends to confer an unpleasant quality.

In functional neuroimaging studies of the healthy brain, the neural processes that support object-level analysis of musical sources (instruments) and patterns (melodies) have been linked to separable but partly overlapping neural networks.^22^ The network that subserves perceptual analysis of timbre includes bilateral temporo-parietal, right anterior temporal and insular cortices^39-41^; the neural substrates of familiar musical instrument recognition (semantic processing of timbres) are not well defined but are likely to inhere in modulation of activity within the wider timbre processing network and premotor cortices, including supplementary motor area (SMA).^41-46^ Canonical melody recognition (semantic processing of melodies) is mediated by a more anterior network encompassing inferior frontal gyrus (IFG), anterior temporal lobe, insula, and SMA,^47-52^ while apperceptive processing of transposed melodies has been shown to engage a bilateral temporo-parietal network overlapping areas involved in the perceptual analysis of timbres.^53-56^ Dissonant melodies engage limbic mechanisms involved in musical emotion coding, including insula, hippocampus, parahippocampal gyrus and amygdala, as well as temporal pole.^37^

Taking a clinical perspective, musical object patterns (melodies) have been more widely addressed than musical object sources (instruments and their timbres) in dementia.^22^ While not previously demonstrated, a deficit of melody apperception might be anticipated in AD, given that apperceptive impairment in AD is well documented for visual objects^9^ and has also been described for non-musical sounds^57^; impaired apperceptive processing of environmental noises has also been reported in svPPA.^11^ Based on melody familiarity and recognition paradigms, musical semantic memory has generally been found to be spared in AD^23,58-60^; this contrasts with the finding of impaired musical episodic memory in this disease.^59^ The relations between musical semantic and episodic memory in AD are of considerable interest,^49^ as the processing of unfamiliarity (novelty) in music (as for other sensory stimuli) engages a neural mechanism targeted by AD pathology at the interface of semantic and episodic memory systems.^59,61-63^ In svPPA, musical semantic (melody recognition) performance is variable.^23,35,64-67^ The basis for selective sparing of musical semantic capacity despite impairment of other semantic domains in some individuals with svPPA is not clear and might reflect the nature of musical object concepts or differentiated neuroanatomical mechanisms.^35,67^ Although less well studied, perceptual processing of acoustic timbral properties relevant to musical instruments (discrimination of spectral shapes and spectrotemporal ripples) has largely been found to be intact in AD and svPPA.^11,57,68,69^ Deficits of musical instrument recognition have been documented in svPPA^35^ and its right temporal lobe syndromic analogue,^70^ corroborating the finding of timbral deficits after anterior temporal lobectomy.^71,72^ While the processing of universal emotions as instantiated in music has been addressed in AD and FTD syndromes,^22,73-75^ there is little information about the processing of emotional attributes specific to musical objects, such as dissonance, with variable findings in patients with FTD versus cognitively well older listeners.^24,76,77^ The musical phenotypes of AD and FTD syndromes map broadly onto known disease-related atrophy signatures and intersect the network anatomy of the healthy musical brain.^22^ In particular, musical semantic and emotion processing profiles have been correlated with overlapping profiles of regional grey matter atrophy in anterior temporal and fronto-subcortical circuitry.^65,74,75,78^ However, there have been very few functional neuroanatomical studies of music processing in AD and FTD syndromes,^59,76^ and the neural mechanisms that sustain musical object processing and sensory object processing more generally in these diseases remain poorly defined.

Here, we addressed musical object analysis as a model for sensory object processing in AD and FTD syndromes using activation functional MRI (fMRI). We aimed to exploit the modular and hierarchical nature of musical object processing and to define the neural mechanisms that encode musical objects corresponding to sources (instrument timbres) and patterns (melodies) in these canonical dementia syndromes. Monophonic melodies were manipulated to assess different dimensions of object processing: detection, feature perception, semantic familiarity, and apperception or object constancy (here, the processing of familiar melodies containing interpolated timbre changes). A further stimulus manipulation contrasted dissonant versus consonant melodies to assess a core emotional attribute of musical objects. We studied patients with AD and temporal lobe variant FTD (tvFTD) relative to age-matched cognitively healthy volunteers (HV). The tvFTD group included patients with predominantly left or right temporal lobe atrophy, syndromically presenting as either svPPA or behavioural variant FTD—semantic deficits are a core feature of the recently recognized, albeit incompletely defined, syndrome of right temporal lobe atrophy, thus lying on a phenotypic continuum with svPPA.^79-81^ To contextualize the fMRI findings, we assessed behavioural correlates of the stimulus manipulations using post-scan neuropsychological tests and disease-related atrophy profiles using voxel-based morphometry (VBM), comparing the syndromic and HV groups.

Based on available evidence,^26,39-51,53^ we hypothesized that timbre change and melody apperception would activate a posteriorly directed temporo-parietal network; semantic processing of melodies and instruments would activate a more anterior network including temporal pole, supplementary motor and inferior frontal cortices; and dissonance perception would engage insula, mesial temporal and subcortical limbic structures. We further hypothesized that patients with AD and tvFTD would have activation profiles similar to cognitively healthy older listeners during timbre change processing, but differential and separable syndromic profiles for semantic familiarity of instruments and melodies and melody constancy, within the cerebral networks mediating these processes in healthy individuals.

## Materials and methods

### Participants

Participants were recruited from the specialist Cognitive Disorders Clinic at the National Hospital for Neurology and Neurosurgery (NHNN) and the University College London (UCL) Dementia Research Centre database. Thirty-five patients (20 AD, 15 tvFTD; mean age 70.3 years, standard deviation 8.4 years) and 25 HV with no history of significant neurological or psychiatric illness (mean age 69.5 years, standard deviation 6.8 years) were included. All patients with AD had a compatible clinical syndrome. Eleven AD patients underwent a lumbar puncture, and all had a CSF biomarker profile (reduced amyloid beta 42:40 ratio and/or elevated total tau or phosphor-Tau 181) consistent with AD pathology, based on local laboratory reference ranges. Inclusion in the tvFTD group was based on the neuroanatomical finding of disproportionate focal anterior temporal lobe atrophy plus a compatible clinical syndrome: 11 patients presented with predominantly left anterior temporal lobe atrophy and fulfilled diagnostic criteria for svPPA at presentation, while the remaining four had predominantly right anterior temporal lobe atrophy and fulfilled criteria for behavioural variant FTD (see Fig. S1). All patients met consensus diagnosis criteria for their syndromic diagnosis^82-84^ of mild to moderate severity and had compatible structural brain MRI findings without significant cerebrovascular burden. No participant had a history of clinically relevant otological disease. All participants completed questionnaires detailing their demographics and musical experience (years of formal musical training and hours of music listening per week) and underwent general neuropsychological assessment and audiometry to assess peripheral hearing following standard procedures (see Table 1 and Supplementary material).

**Table 1:** Demographic, clinical and neuropsychological characteristics of diagnostic groups

Characteristic	HV	AD	tvFTD	Omnibus test
General				
No.	25	20	15^a^	NA
Age (years)	69.5 (6.8)	73.9 (7.0)	65.5 (7.9)	*X^2^*(2) = 10.0, *P* = 0.007^b^
Sex (F:M)	16:9	8:12	6:9	*P* = 0.209
Highest education level^c^	3.6 (1.9)	3.2 (1.9)	4.1 (1.3)	*X^2^*(2) = 2.2, *P* = 0.340
Handedness (R:L)	23:2	19:1	10:5	*P* = 0.051
Symptom duration (years)	NA	5.0 (2.1)	5.3 (3.6)	*W* = 158.5, *P* = 0.788
MMSE (/30)	28.7 (1.4)^d^	***20.0*** (***5.8)***	**23.7** (**8.3)**	*X^2^*(2) = 32.7, *P* < 0.001
Auditory function				
PTA BEA (dB)^e^	16.7 (6.3)	23.8 (12.2)	17.9 (14.9)	*X^2^*(2) = 5.1, *P* = 0.079
Pitch change detection (/20)^f^	19.7 (0.8)^g^	***17.6*** (***3.1)***^g^	19.7 (1.3)	*X^2^*(2) = 12.8, *P* = 0.002
Musical background				
Musical training (years)	3.6 (4.7)	1.9 (2.3)^d^	3.7 (5.2)	*X^2^*(2) = 0.2, *P* = 0.901
Music listening (hours/week)	13.2 (12.2)	9.4 (11.7)^d^	8.3 (8.2)	*X^2^*(2) = 2.5, *P* = 0.294
General neuropsychological assessment				
*Executive*				
WASI-MR (/32)	26.1 (2.4)	***14.5*** (***8.0)***	25.5 (8.5)	*X^2^*(2) = 30.2, *P* < 0.001
TMT A (/150 s)	28.2 (9.0)^h^	***73.1*** (***41.2)^i^***	44.7 (46.7)^i^	*X^2^*(2) = 21.0, *P* < 0.001
TMT B (/300 s)	61.1 (15.4)^h^	***222.7*** (***84.2)^i^***	88.0 (74.7)^i^	*X^2^*(2) = 27.4, *P* < 0.001
Phonological Fluency	18.4 (4.9)^j^	**10.5** (**7.8)**^g^	**11.2** (**5.7)**^k^	*X^2^*(2) = 12.8, *P* = 0.002
Categorical Fluency	24.6 (4.1)^j^	**9.8** (**6.2)^g^**	**12.9** (**7.3)^k^**	*X^2^*(2) = 27.3, *P* < 0.001
TEA Elevator Counting (/6)	6.0 (0.0)^k^	**5.1** (**1.8)**	5.7 (0.6)^i^	*X^2^*(2) = 9.4, *P* = 0.009
TEA Elevator Distraction (/9)	7.3 (2.7)^i^	***1.7*** (***2.1)*^k^**	7.5 (2.8)^i^	*X^2^*(2) = 25.3, *P* < 0.001
CPAL (/24)	20.6 (2.2)^l^	**3.9** (**4.2)^m^**	**10.3** (**8.8)^g^**	*X^2^*(2) = 25.6, *P* < 0.001
GDA (/24)	14.8 (4.7)^j^	***5.0*** (***4.7)*^g^**	11.6 (6.1)^k^	*X^2^*(2) = 19.8, *P* < 0.001
*Working memory*				
WMSR-DS Forward (/12)	9.5 (1.7)	**7.4** (**2.5)**	8.8 (1.9)	*X^2^*(2) = 9.5, *P* = 0.009
WMSR-DS Backward (/12)	7.6 (2.5)	***4.7*** (***1.7)***	7.1 (1.9)	*X^2^*(2) = 16.5, *P* < 0.001
Spatial Span Forward (/12)	7.9 (2.2)	***5.6*** (***2.1)***	7.5 (1.6)^k^	*X^2^*(2) = 14.0, *P* < 0.001
Spatial Span Backward (/12)	7.8 (1.4)	***4.4*** (***2.7)***	8.4 (1.9)^k^	*X^2^*(2) = 23.7, *P* < 0.001
*Language*				
BNT (/30)^e^	29.1 (1.2)^j^	**20.6** (**6.8)^k^**	**12.7** (**10.0)^d^**	*X^2^*(2) = 34.2, *P* < 0.001
BPVS (/150)	145.4 (4.6)^n^	140.2 (16.4)	***119.0*** (***34.4)***^d^	*X^2^*(2) = 8.7, *P* = 0.013
*Visual function*				
JoLO (/30)^e^	27.5 (2.8)	***20.0*** (***7.9)^i^***	26.4 (8.2)^d^	*X^2^*(2) = 12.9, *P* = 0.002
VOSP-OD (/20)	18.4 (1.4)	**16.2** (**2.3)**^d^	**17.1** (**2.0)**	*X^2^*(2) = 14.1, *P* < 0.001
*Socio-emotional processing*				
Emotional Hexagon (/24)	21.4 (1.9)	**17.7** (**3.4)^g^**	**17.4** (**4.4)**	*X^2^*(2) = 16.9, *P* < 0.001
CFMT-E (/18)	17.5 (1.2)	***11.8*** (***3.7)***^d^	**16.3** (**1.5)**	*X^2^*(2) = 30.8, *P* < 0.001
ECAS—Social Cognition Screen (/12)	11.8 (0.6)	***9.8*** (***3.1)***^k^	11.8 (0.6)^d^	*X^2^*(2) = 10.1, *P* = 0.006

Mean (standard deviation) values are provided for continuous variables; counts are given for categorical variables. Omnibus test statistics for overall group effects are included, where appropriate. Maximum scores for tasks are indicated in parentheses where available. Bold indicates significantly worse performance than the HV group; italics indicate significantly worse performance than the other patient group. A Wilcoxon signed-rank test was used to assess patient-group differences in symptom duration. Kruskal-Wallis ANOVA and Dunn’s tests were used to assess group differences in all other continuous variables. Fisher’s exact test was used for categorical variables. AD, patient group with Alzheimer’s disease; ANOVA, analysis of variance; BNT, Boston Naming Test; BPVS, British Picture Vocabulary Scale; CFMT-E, Cambridge Face Memory Test—Encoding subtask; CPAL, Camden Paired Associates Learning; dB, decibels; DRC, Dementia Research Centre; ECAS, Edinburgh Cognitive and Behavioural Screen; GCSE, General Certificate of Secondary Education; GDA, Graded Difficulty Arithmetic; HV, healthy volunteer; JoLO, Judgement of Line Orientation; L, Left; MMSE, Mini-Mental State Examination; NA, Not Applicable; PTA BEA, pure tone audiometry better ear average; R, Right; TEA, Test of Everyday Attention; TMT, Trails Making Test; tvFTD, patient group with temporal variant frontotemporal dementia; UK, United Kingdom; VOSP-OD, Visual Object Space Perception—Object Decision subtask; WASI-MR, Wechsler Adult Scale of Intelligence—Matrix Reasoning subtask; WMSR-DS, Wechsler Memory Scale Revised—Digit Span subtask.

^a^Eleven patients had semantic variant primary progressive aphasia with more marked left than right temporal lobe atrophy, and four had behavioural variant frontotemporal dementia with more marked right than left temporal lobe atrophy (see Fig. S1).

^b^The AD group was significantly older than the HV and tvFTD groups.

^c^Highest level of education (UK system) was measured on an ordinal scale where 0 = less than O-levels/GCSE, 1 = O-levels/GCSE, 2 = A-levels, 3 = some university, or technical degree, 4 = Bachelor’s degree, 5 = Postgraduate degree.

^d^One participant missing.

^e^Adapted version used, see Supplementary material for details.

^f^Bespoke task described in Supplementary Materials.

^g^Three participants missing.

^h^Six participants missing.

^i^Four participants missing.

^j^Seven participants missing.

^k^Two participants missing.

^l^Nine participants missing.

^m^Five participants missing.

^n^Based on five participants (performance on this test similar to a historical HV cohort studied at the DRC).

All participants provided written informed consent. This study was approved by the UCL and NHNN Joint Research Ethics Committees, aligning with the Declaration of Helsinki guidelines.

### Experimental stimuli and conditions

**Figure 1:**
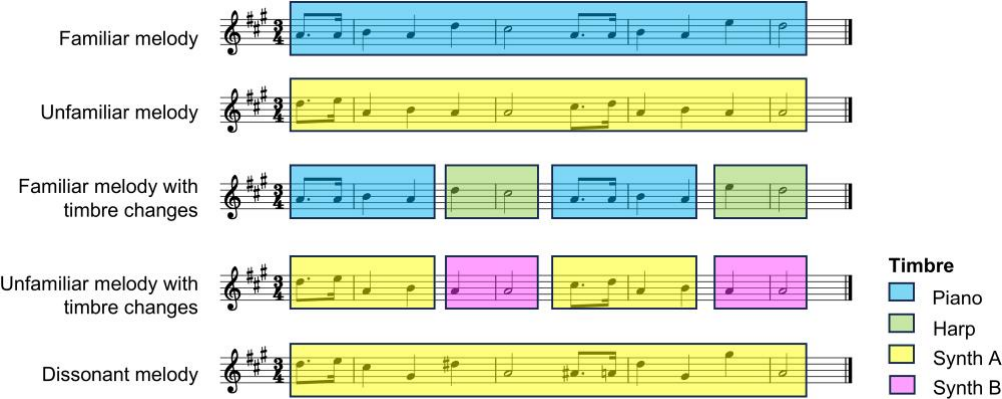
**Schematic of experimental conditions.** The figure diagrams trials representing each of the experimental conditions in the fMRI experiment; all examples shown are based on the melody ‘*Happy Birthday’*. The legend (bottom right) indicates the timbre in which the melody was presented; Synth A and Synth B refer to the two artificial timbres (see main text). The timbres assigned to each condition were balanced across all trials (see Table S2). In the unfamiliar melody condition, the pitch sequence was reversed, but the rhythmic structure stayed the same. In the dissonant melody condition, pitches of the unfamiliar melody condition were altered so that they violate the key signature. In the conditions containing timbre changes, the transitions between timbres were timed to minimize melody identification from any of the fragments in a single timbre.

The music stimuli comprised monophonic melodies, created as digital audio files using MuseScore®4 software with four different instrument timbres: two natural (familiar) instruments (piano, harp) and two novel, artificial (unfamiliar) instruments (MuseScore®4 ‘square’ [Synth A] and ‘brightness’ [Synth B] synthesisers), allowing for the manipulation of instrument familiarity. There were five active experimental conditions in an unbalanced 2 × 2 factorial design: familiar melodies without timbre changes (FM), unfamiliar melodies without timbre changes (UM), familiar melodies with timbre changes (FMTC), unfamiliar melodies with timbre changes (UMTC), and unfamiliar dissonant melodies (DM). A baseline silence condition was also included. These conditions allowed us to capture principal musical object processing contrasts: auditory stimulation (all music conditions > silence), perceptual discrimination of musical features (main effect of timbre change: FMTC + UMTC > FM + UM), musical semantic processing of familiar versus unfamiliar melodies (main effect of melody familiarity: FM + FMTC > UM + UMTC), and the processing of familiar versus unfamiliar melodies in the presence of interpolated timbre changes corresponding to apperceptive object constancy (interaction effect of melody constancy: [FMTC > UMTC] > [FM > UM]). We additionally assessed the reverse of melody familiarity (melody novelty: UM + UMTC > FM + FMTC), semantic processing of musical sources (instrument familiarity: UM + UMTC with natural instruments > UM + UMTC with artificial instruments) and dissonance (DM > UM). Stimulus examples are schematized in Fig. 1, with audio files in the Supplementary material.

For the FM and FMTC conditions, 34 melodies that were highly familiar to the general British older adult population were selected based on an initial musical norming study (see Supplementary material). A range of genres (Western classical, pop, Christmas, nursery, and traditional) and emotional valences were represented. Melodies with native keys were kept in their original key signature, and a range of key signatures was used across the stimulus set.

The UM and UMTC conditions comprised novel, pseudo-reversed versions of the melodies used in the FM condition, such that the pitch sequence was reversed, but the rhythmic structure was the same. An experienced musician (LBC) assessed each novel melody and made small modifications to the notes where necessary, to ensure that the corresponding familiar melody could not be detected in its reversed form. Similar procedures have been used in previous music work.^23,47^ Stimuli in the DM condition were created based on the UM melodies; the pitches of several notes were changed so that they violated the key signature of the rest of the melody.

In the FMTC and UMTC conditions, all trials contained two timbre changes within the melody to minimize identification of familiar melodies from any single timbral section. The positions of changes within trials were slightly varied to avoid expectation effects. The type of timbre (natural or artificial) remained the same within a single trial. All timbres were represented equally across the experiment (Table S2), and the familiar melody trials employing each timbral carrier were balanced for general musical characteristics (Table S3).

Audio files were processed using a custom MATLAB® script to fix intensity (root mean square level) across the stimulus set. All sound files were trimmed digitally using Audacity software to remove trailing silence, ensuring they were exactly 7.9 s in duration, to fit the timing constraints of a sparse sampling fMRI acquisition.

### Experimental protocol

#### Brain image acquisition

During scanning, stimuli were presented binaurally via MRI-compatible headphones (www.mr-confon.de) from a laptop running a bespoke stimulus presentation script in MATLAB® version 2023a with PsychoToolbox-3. The loudness was adjusted prior to the experiment during the presentation of two practice trials so that it was comfortable for each participant. Each participant was instructed to lie still and listen to the melodies; there was no in-scanner task. Participants were also asked to fixate on a black cross in the centre of a projector screen during music listening to reduce extraneous eye movements.

Trials were presented in a fixed pseudo-randomized order such that the unfamiliar (pseudo-reversed) version of a familiar melody never immediately followed the familiar version. In total, 184 trials (5 × 34 melody stimuli plus 14 silence trials) were delivered across four runs.

Whole-brain images were acquired using a 64-channel radiofrequency head coil on a Siemens Prisma 3-Tesla MRI scanner at the NHNN. To avoid interactions of scanner acoustic noise and auditory stimulus presentation, we used a sparse sampling design,^85^ whereby auditory stimuli were presented interleaved with scanner noise during an eight-second silent interval between image acquisitions. fMRI data were acquired with a gradient-echo planar imaging sequence. The first fourteen participants received a multi-band acquisition with an acceleration factor of 2 (TR [repetition time] = 9700 ms, TE [echo time] = 29.2 ms, FOV [field of view] = 202 mm). The remaining participants received a non-multiband acquisition (TR = 11 030 ms; TE = 30 ms; FOV = 192 mm). The voxel size (resolution) was 2.1 × 2.1 × 2.0 mm for the multiband sequence and 2.0 × 2.0 × 2.0 mm for the non-multiband sequence. A T1-weighted structural scan (MPRAGE [Magnetisation Prepared Rapid Gradient Echo] sequence, TR = 2000 ms, TE = 2.93 ms, flip angle = 8°, 208 sagittal slices, 1.1 × 1.1 × 1.1 mm voxels) was also acquired for each participant. The scanning session lasted approximately one hour.

#### Post-scan behavioural tests

After the fMRI session, participants completed behavioural tests to assess their sensitivity to the musical stimulus manipulations (timbre change, melody and instrument familiarity) heard during scanning. In each test, the task was based on a two-alternative forced-choice response procedure. To assess timbre change detection, eight UM stimuli and the corresponding eight UMTC stimuli were presented; on each trial, the participant decided whether the instrument playing the melody stayed the same or changed. To assess melody familiarity, all 68 trials in the FM and UM conditions were presented; on each trial, the task was to decide whether the melody was a famous tune. To assess melody constancy (apperception), all 68 trials in the FMTC and UMTC conditions were presented; on each trial, the task was again to decide whether the melody was a famous tune. The melody constancy and melody familiarity tasks were administered in a counterbalanced order at least one hour apart to avoid any priming effects. To assess instrument familiarity, 16 trials from the UM condition were presented, equally representing natural and artificial timbres; on each trial, the task was to decide if the instrument (timbre) heard was natural or artificial. To assess dissonance detection, eight DM trials and the corresponding UM trials were presented; on each trial, the task was to decide if the melody sounded out of tune or had wrong notes.

We also administered a test to assess pitch changes as an index of performance on an auditory task, elementary to music perception. This test followed a previously described procedure (see Supplementary material), and participants’ scores were included as a covariate in the analysis of musical task performance.

### Data analyses

#### Brain image pre-processing and analysis

All structural and functional images were visually inspected for quality control (see Supplementary material). Pre-processing and analysis steps were carried out using SPM12 (Department of Imaging Neuroscience, https://www.fil.ion.ucl.ac.uk/spm) and MATLAB® version 2014b. Structural brain images were compared between each patient group and the HV group in a VBM analysis (see Supplementary material). For the functional neuroanatomical data, the first three volumes of the dataset were discarded to account for T1 equilibrium effects. The remaining images were realigned to the first image of the series to account for small head movements. The data were normalized to Montreal Neurological Institute space, and spatial smoothing was applied using a Gaussian kernel with a full width at half maximum of 4 mm. Pre-processed functional images were entered into a first-level analysis for each participant. Each experimental condition was included as a separate regressor, modelled as a boxcar across the duration of each trial and convolved with the canonical hemodynamic response function. Head motion parameters from realignment were modelled as covariates of no interest; silence trials were modelled as an implicit baseline. We compared experimental conditions using a series of t-contrasts (specified above). First-level contrast images for each participant were entered into a second-level group analysis with covariates of age (mean-centred) and scanner protocol. One-way ANOVAs were employed to determine where in the brain each of the first-level t-contrasts elicited activation.

For each first-level contrast, we initially assessed the pooled effect across participant groups (using t-contrasts including all participant groups; see Table S5 and Fig. S5) and the interaction with participant group (using F-contrasts; see Table S6 and Fig. S6) over the whole brain, at an uncorrected threshold of *P* < 0.001.

**Figure 2:**
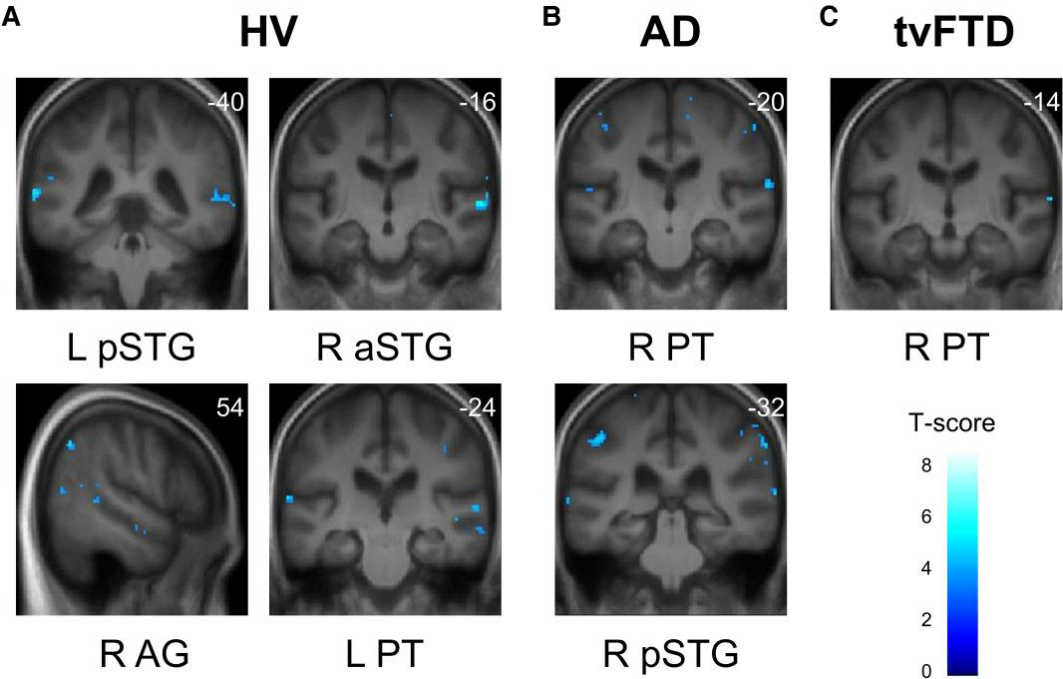
**Musical object processing fMRI profiles: significant activation for perceptual processing within diagnostic groups.** The figure shows statistical parametric maps of brain activation in the group-level fMRI analysis for the timbre change t-contrast within **(A)** the healthy volunteer (HV) group [*n* = 25], **(B)** the Alzheimer’s disease (AD) group [*n* = 20] and **(C)** the temporal variant frontotemporal dementia (tvFTD) group [*n* = 15] (see also Table 2). T-contrasts were also used to assess group activations at the second level. Activations are overlaid on coronal or sagittal sections of the group mean structural brain image and thresholded at an uncorrected level of *P* < 0.001 for display purposes; local maxima are all significant at *P* < 0.05 after family-wise error correction for multiple voxel-wise comparisons within the pre-specified neuroanatomical region of interest (see text and Fig. S2), and no other significant activations were identified at the prescribed threshold. The associated coordinate in Montreal Neurological Institute space is indicated in the upper right-hand corner of each brain image; the left cerebral hemisphere is displayed on the left of each coronal section. The colour bar codes voxel-wise T-scores. AG, angular gyrus; a/pSTG, anterior/posterior superior temporal gyrus; L, left; PT, planum temporale; R, right;.

**Figure 3:**
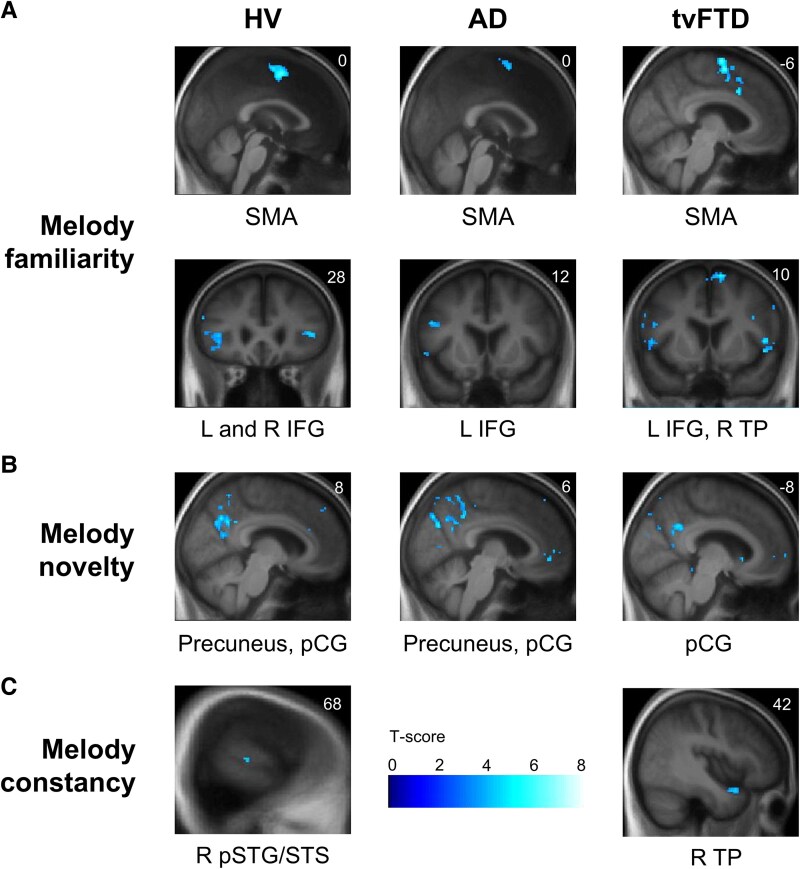
**Musical object processing fMRI profiles: significant activation for semantic and apperceptive processing within diagnostic groups**. The figure shows statistical parametric maps of brain activation in the group-level fMRI analysis for the melody processing contrasts within each diagnostic group (see also Table 2) for the (**A**) melody familiarity, (**B**) melody novelty and (**C**) melody constancy contrasts (see main text). T-contrasts were used to assess comparisons between experimental conditions at the first level and group activations at the second level. *n* = 25, 20, 15 for the HV, AD, and tvFTD groups, respectively. Activations are overlaid on coronal or sagittal sections of the group mean structural brain image and thresholded at an uncorrected level of *P* < 0.001 for display purposes; local maxima are all significant at *P* < 0.05 after family-wise error correction for multiple voxel-wise comparisons within the pre-specified neuroanatomical region of interest (see text and Fig. S2). No other significant activations for melody processing or instrument familiarity contrasts were identified at the prescribed threshold. The associated coordinate in Montreal Neurological Institute space is indicated in the upper right-hand corner of each brain image; the left cerebral hemisphere is displayed on the left of each coronal section. The colour bar codes voxel-wise T-scores. AD, patient group with Alzheimer’s disease; HV, healthy volunteer group; IFG, inferior frontal gyrus; L, left; pCG, posterior cingulate gyrus; R, right; SMA, supplementary motor area; pSTG/STS, posterior superior temporal gyrus/sulcus; TP, temporal pole; tvFTD, patient group with temporal variant frontotemporal dementia.

**Figure 4:**
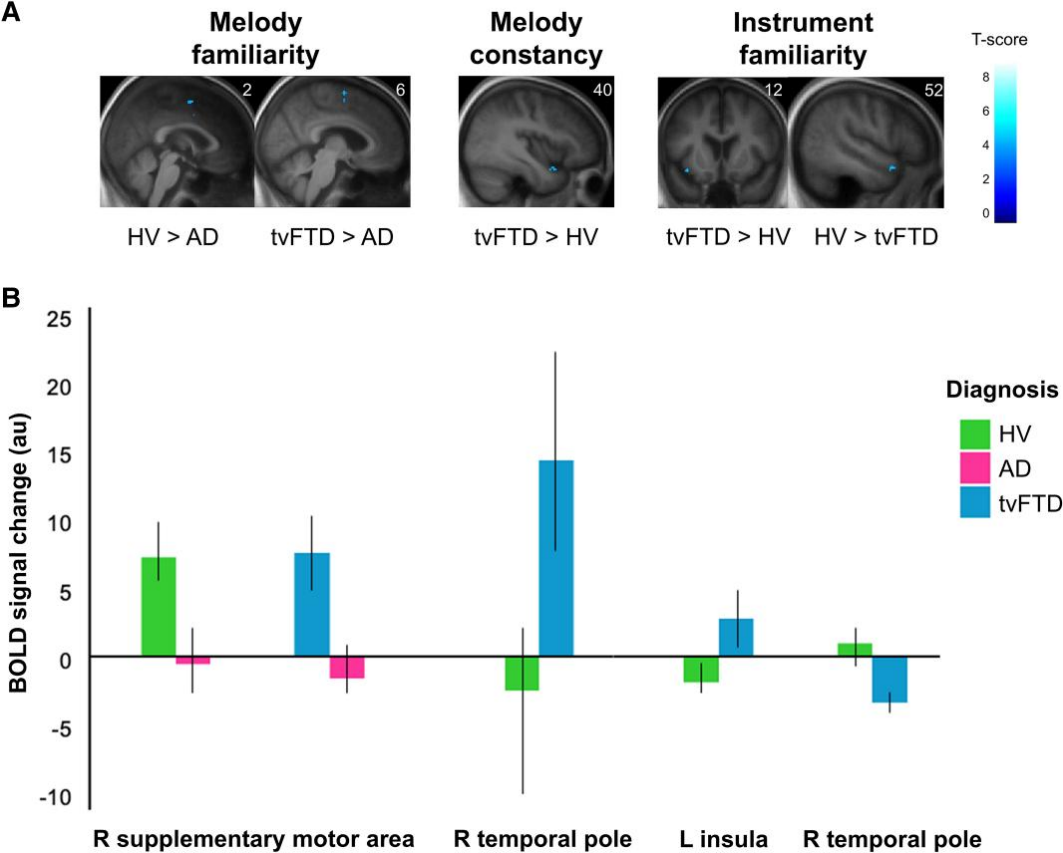
**Musical object processing fMRI profiles: significant activation between diagnostic groups. (A)** The top panel show statistical parametric maps of significant brain activation differences between diagnostic groups in the fMRI analysis for music processing contrasts (see also Table 2). T-contrasts were used to assess comparisons between experimental conditions at the first level and group activations at the second level. *n* = 25, 20, 15 for the HV, AD, and tvFTD groups, respectively. Activations are overlaid on coronal or sagittal sections of the group mean structural brain image and thresholded at an uncorrected level of *P* < 0.001 for display purposes; local maxima are all significant at *P* < 0.05 after family-wise error correction for multiple voxel-wise comparisons within pre-specified anatomical regions of interest (see text and Fig. S2), and no other significant activations were identified at the prescribed threshold. The associated coordinate in Montreal Neurological Institute space is indicated in the upper right-hand corner of each brain image; the left cerebral hemisphere is displayed on the left of the coronal section. The colour bar codes voxel-wise T-scores. **(B)** The bottom panel shows group blood-oxygen-level-dependent (BOLD) signal changes for each contrast at voxels with peak significant between-group activation differences; the brain regions containing peak activation differences are indicated here and correspond to peak voxel coordinates in Table 2. Changes are referenced to a notional ‘zero’ level representing the global mean activation; error bars represent 90% confidence intervals around the group mean BOLD signal change. AD, patient group with Alzheimer’s disease; au, arbitrary units; BOLD, blood-oxygenation-level-dependent; HV, healthy volunteer group; L, left; R, right; tvFTD, patient group with temporal variant frontotemporal dementia.

We followed up with analyses within each participant group and compared each patient group to the HV group using two-sample *t*-tests; where the comparison with HV was significant, differences were also assessed between patient groups. For these group-wise analyses, we performed small volume corrections; this mass univariate approach limits the search for significant voxels to pre-specified neuroanatomical regions of interest. We report peak-level activations thresholded at *P* < 0.05 after family-wise error (FWE) correction for multiple voxel-wise comparisons within the pre-specified regions of interest for each hemisphere. The regions were derived from the Harvard-Oxford atlas^86^ in FSL View^87^ (Fig. S2), selected based on prior evidence of involvement in the process of interest and comprised: for all music conditions over silence (auditory stimulation), Heschl’s gyrus; for the main effect of timbre change, planum temporale, superior temporal gyrus (STG), inferior parietal lobe^39-41^; for the main effect of melody familiarity, temporal pole, SMA, and IFG^47-51,59^; for the interaction effect of melody constancy, STG, inferior parietal lobe, temporal pole, and IFG^53^; for melody novelty, precuneus and posterior cingulate gyrus^59^; for instrument familiarity, temporal pole and insula^41-46^; and for dissonance, insula, amygdala and frontal operculum.^37^ An uncorrected significance level of *P* < 0.001 over the whole brain was used to generate statistical parametric maps shown in Figs 2–4, Figs S4–S6.

For the contrasts where we observed significant differences in either patient group versus the HV group, we conducted additional analyses to examine the effect of behavioural task performance on brain activation (see Supplementary material).

#### Behavioural data analysis

Hit and false alarm rates on each post-scan behavioural task were calculated to derive A-prime, a non-parametric summary measure of sensitivity to stimulus parameters, in an adapted Excel workbook.^88^ A continuity correction was applied to account for hit or false alarm rates of exactly 0 or 1. Using R version 4.3.2 and the ‘rfit’ package,^89^ we employed a rank-sum analysis of covariance to assess group differences in A-prime for each musical post-scan task, adjusted for age and score on the pitch change detection task. This non-parametric approach was employed because the normality assumption, assessed using Shapiro-Wilk tests, was violated for all post-scan tasks. A statistical significance threshold of *P* < 0.05 was used for all tests.

## Results

### General characteristics of participant groups

General demographic, clinical, and neuropsychological profiles of participant groups are summarized in Table 1.

There was a main effect of group on age (*X^2^*(2) = 10.0, *P* = 0.007); post hoc comparisons revealed that the AD group was significantly older than the HV (*z*(2) = −3.1, *P* = 0.001) and tvFTD (*z*(2) = −2.1, *P* = 0.041) groups, thus age was included as a covariate in further analyses. There were no significant group differences in sex (*P* = 0.209), peripheral hearing function (*X^2^*(2) = 5.1, *P* = 0.079), educational attainment (*X^2^*(2) = 2.2, *P* = 0.340), years of musical training (*X^2^*(2) = 0.2, *P* = 0.901), or current music listening (*X^2^*(2) = 2.5, *P* = 0.294) across groups. There was a main effect of group on Mini-Mental State Examination score (*X^2^*(2) = 32.7, *P* < 0.001): both patient groups performed worse than the HV group (AD: *z*(2) = −5.71, *P* < 0.001; tvFTD: *z*(2) = −2.7, *P* = 0.006), and the AD group performed worse than the tvFTD group (*z*(2) = 2.4, *P* = 0.015). There was no significant difference in symptom duration between the patient groups (*W* = 158.5, *P* = 0.788).

### Neuroanatomical data

The VBM analysis demonstrated the anticipated profiles of regional grey matter atrophy in the AD and tvFTD groups relative to the HV group (Table S4, Fig. S3). There were no significant differences in atrophy patterns between the two patient groups.

For the fMRI analysis, results for the pooled analysis across groups and for the interaction effect of group with each principal contrast are presented in Tables S5 and S6. The activation profiles in these analyses were in line with our prior anatomical hypotheses and selection of regions of interest, revealing a predominantly posteriorly directed temporo-parietal network engaged in processing timbre change and melody constancy, and more anterior temporal and prefrontal areas engaged in semantic processing of melodies and instruments. Given these findings, and in light of the neuroanatomical heterogeneity of the syndromic groups here, we focus below on the within- and between-group analyses for prespecified regions of interest.

The effect of any auditory stimulation (versus silence) manifested in activation of Heschl’s gyrus bilaterally for each diagnostic group (Table S7; Fig. S4); there were no significant differences in brain activation between groups.

For the principal contrasts addressing musical object processing, distinct functional neuroimaging associations were identified within and between diagnostic groups, presented in Table 2. Statistical parametric maps of significant contrasts assessing perceptual (timbre change) processing within each participant group are presented in Fig. 2; maps of significant contrasts assessing semantic and apperceptive processing within each participant group are presented in Fig. 3; maps of significant between-group contrasts and associated peak blood-oxygen-level-dependent (BOLD) signal changes are shown in Fig. 4.

**Table 2:** fMRI of music processing: significant contrasts within and between diagnostic groups

Analysis	Groups	Region	Side	Cluster size (voxels)	Peak coordinates (mm)	Z-score	P_FWE_-value
Timbre change	HV	Posterior superior temporal gyrus	L	17	−66, −40, 8	4.26	0.006
		Anterior superior temporal gyrus	R	8	64, −16, 0	4.23	0.007
		Angular gyrus	R	8	54, −58, 40	4.19	0.040
		Planum temporale	L	7	−66, −24, 12	4.09	0.011
	AD	Planum temporale	R	8	66, −20, 14	4.02	0.012
		Posterior superior temporal gyrus	R	5	68, −32, 10	3.89	0.031
	tvFTD	Planum temporale	R	3	68, −14, 6	3.92	0.027
Melody familiarity	HV	Supplementary motor area	L	181	0, 2, 58	5.20	<0.001
			R	94	2, 0, 56	5.09	<0.001
		Inferior frontal gyrus	L	235	−50, 22, 24	4.72	0.002
			R	9	42, 28, 6	4.08	0.027
		Temporal pole	L	14	−54, 12, −4	4.42	0.003
	AD	Inferior frontal gyrus	L	16	−48, 12, 26	4.07	0.020
		Supplementary motor area	L	29	0, 4, 62	4.06	0.014
	tvFTD	Supplementary motor area	L	202	−6, −4, 70	5.39	<0.001
			R	98	4, 0, 64	4.70	0.001
		Inferior frontal gyrus	L	104	−52, 18, 14	4.40	0.007
		Temporal Pole	R	26	48, 10, −6	4.25	0.008
	HV > AD	Supplementary motor area	R	5	2, 0, 56	3.76	0.045
	tvFTD > AD	Supplementary motor area	R	6	6, −2, 66	3.89	0.027
Melody constancy	HV	Posterior superior temporal gyrus/sulcus	R	3	68, −28, 2	3.85	0.036
	tvFTD	Temporal pole	R	30	42, 12, −22	4.05	0.019
	tvFTD > HV	Temporal pole	R	12	40, 10, −22	4.05	0.019
Melody novelty	HV	Precuneus	R	417	6, −62, 36	5.25	<0.001
			L	265	−8, −60, 52	4.59	0.006
		Posterior cingulate gyrus	R	26	8, −48, 32	3.97	0.017
			L	8	0, −52, 32	3.86	0.030
	AD	Precuneus	R	138	6, −70, 30	4.72	0.003
			L	136	−8, −70, 30	4.57	0.006
		Posterior cingulate gyrus	R	12	6, −44, 38	4.10	0.010
			L	17	−2, −38, 46	4.01	0.016
	tvFTD	Posterior cingulate gyrus	L	40	−8, −54, 32	4.25	0.006
Instrument familiarity	HV > tvFTD	Temporal pole	R	9	52, 12, −20	4.04	0.020
	tvFTD > HV	Anterior insula	L	6	−36, 12, −14	3.95	0.045
Dissonance detection	HV	Amygdala	R	1	28, −6, −22	3.39	0.004

Peak activations are significant (*P* < 0.05) after family-wise error (FWE) correction within pre-specified anatomical regions of interest (see main text). Peak coordinates are in Montreal Neurological Institute standard space. AD, patient group with Alzheimer’s disease; FWE, family wise error; HV, healthy volunteer group; tvFTD, patient group with temporal variant frontotemporal dementia.

For the main effect of timbre change, the HV group showed significant activations in the angular gyrus bilaterally, left posterior STG, planum temporale and right anterior STG. The AD showed right posterior STG activation, and both patient groups showed significant activations in right planum temporale.

For the main effect of melody familiarity, the HV group showed significant activations in SMA and IFG bilaterally, and in left temporal pole. The AD and tvFTD groups both showed significant activations in left SMA and IFG, and the tvFTD group showed additional activation in right SMA and temporal pole; activation in right SMA was significantly greater in the HV and tvFTD groups than in the AD group.

For the interaction between familiarity and timbre change (apperceptive melody constancy), the HV group showed significant activation in right posterior STG and sulcus. The tvFTD group showed activation in right temporal pole which was significantly greater than in the HV group.

For the melody novelty contrast, the HV group showed significant activations in precuneus and posterior cingulate gyrus bilaterally; the AD group also showed bilateral activations in these regions, while the tvFTD group showed activation restricted to left posterior cingulate gyrus. For the instrument familiarity contrast, no significant activations were observed within groups, however the HV and tvFTD groups showed significant between-group differences driven by activation in left anterior insula and deactivation in right temporal pole in the tvFTD group. For the dissonance contrast, the HV group showed significant activation in right amygdala. No other significant within-group activations or between-group activation differences were identified in our pre-specified anatomical regions. Further, we observed no main effect of behaviour nor group by behaviour interaction on brain activation (see Supplementary material).

Comparisons of significant activations with group profiles of regional atrophy (Table S4) revealed no overlap in the AD group. For the tvFTD group, the activations in left planum temporale, IFG and SMA did not coincide with the atrophy map: further, the activations in right temporal pole and left insula (within the zone of atrophy) were increased compared to the HV group (Fig. 4).

### Behavioural data

**Figure 5:**
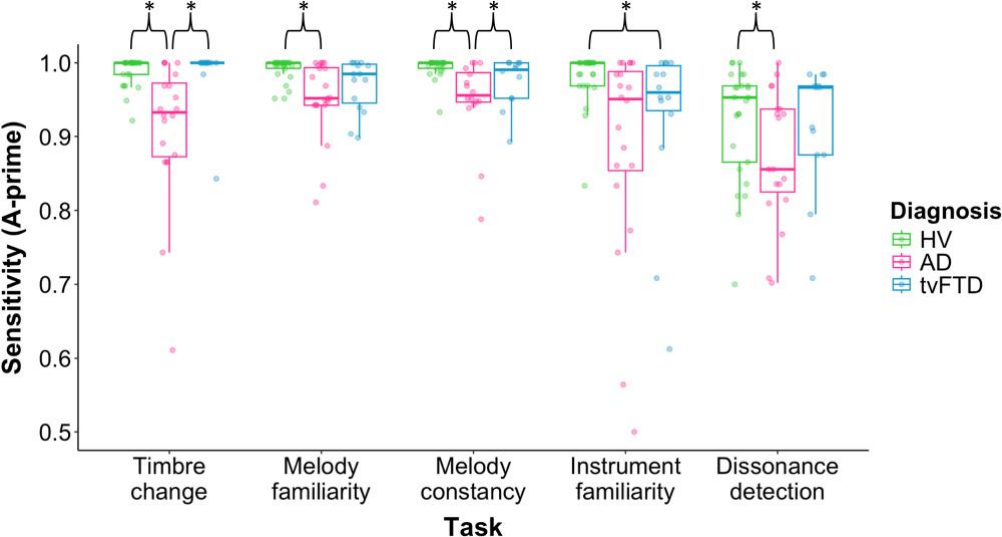
**Group behavioural performance profiles on post-scan music tasks.** Boxplots show the median and interquartile A-prime ranges for each diagnostic group in each of the post-scan musical tasks; individual data points are overlaid. Asterisks (*) indicate a significant difference between groups at *P* < 0.05, after controlling for age and performance on the pitch change detection task using non-parametric rank-sum analyses of covariance. Group sizes were as follows: HV, *n* = 25 (except melody constancy, *n* = 23); AD, *n* = 20 (except melody constancy, *n* = 16; dissonance detection, *n* = 19); tvFTD, *n* = 15 (except timbre change and dissonance, *n* = 13; instrument familiarity, *n* = 14; melody constancy, *n* = 12). AD, patient group with Alzheimer’s disease; HV, healthy volunteer group; tvFTD, patient group with temporal variant frontotemporal dementia.

Performance on the post-scan music tasks for each participant group is shown in Table S8 and Fig. 5. The AD group performed significantly worse than the HV group on timbre change detection (*t*(56) = −6.5, *P* < 0.001), melody familiarity (*t*(56) = −2.4, *P* = 0.022), melody constancy (*t*(56)= −3.9, *P* < 0.001), and dissonance detection (*t*(56) = −2.3, *P* = 0.023). The tvFTD group performed significantly worse than the HV group on instrument familiarity (*t*(56) = −2.3, *P* = 0.025). Comparing syndromic groups, the AD group performed significantly worse than the tvFTD group on timbre change detection (*t*(56) = −5.3, *P* < 0.001) and melody constancy (*t*(56) = −2.2, *P* = 0.032).

## Discussion

Here, we have delineated a hierarchy of functional neuroanatomical substrates for auditory object processing in AD and FTD versus HV, using the paradigm of music. As anticipated, musical sound detection was mediated by a common functional neuroanatomical mechanism centred on Heschl’s gyrus (primary auditory cortex) across diagnostic groups. Overlapping but separable neuroanatomical mechanisms were shown to mediate the analysis of musical sources (instruments) and patterns (melodies), and within these different categories of musical objects, the coding of perceptual features, familiarity and novelty. For the perceptual representation of changing timbres, both syndromic groups and the HV group demonstrated comparable temporo-parietal cortical activation anchored in planum temporale. Semantic familiarity of melodies in both syndromic groups and HV engaged a common network including SMA and IFG, albeit with reduced activation of SMA in AD compared with other groups; novelty comparably engaged postero-medial cortical circuitry anchored in posterior cingulate across groups. Apperceptive coding of melody constancy was associated with activation of the posterior superior temporal cortex in the HV group, but greater activation of the temporal polar cortex in the tvFTD group than in the HV group. Musical instrument familiarity processing was associated with reduced activation of the temporal polar cortex but increased activation of the anterior insula in tvFTD compared to HV. These findings speak to the highly fractionated cognitive and neural organisation of musical and other auditory object processing and associated deficits in AD and tvFTD.^18,35,59,90,91^

The neuroanatomical signatures of musical object processing identified in the HV group distil and extend previous work on object perception in the auditory modality. To the extent that these signatures were shared by the AD and tvFTD groups, our findings further suggest that much of the neural machinery of music processing continues to operate in these dementia syndromes, albeit with often reduced performance (see Fig. 5). Timbre change processing entails the discrimination of musical sources based on perceptual features: planum temporale and posterior STG were identified as cross-diagnostic hubs, in line with the generic role previously attributed to these regions in the parsing and templating of spectrotemporal features,^92-94^ and more specifically the perception of sound source identity^95^ and musical timbre.^96^ The additional inferior parietal and anterior superior temporal activations in the HV group align with the timbre processing network previously delineated in the healthy brain.^39^ Posterior superior temporal cortex also plays a key role in the perceptual coding of pitch patterns as prototypical auditory objects,^97,98^ and in templating and disambiguating different auditory objects,^53-56^ likely accounting for the engagement of this region during apperceptive melody processing (melody perception despite interpolated timbre changes) in the HV group. Indeed, our timbre change contrast also entailed an apperceptive dimension, since timbre changes occurred in the context of continually changing pitch carriers^32^; it is therefore likely that the temporo-parietal correlates of timbre change processing here encompass neural mechanisms for coding timbre constancy, analogous to those coding melody constancy.

Our findings further corroborate previous studies delineating a cross-diagnostic frontotemporal network for musical semantic memory, in which SMA and IFG play integral roles beyond the canonical temporo-polar semantic hub.^47,48,50,51,59,99,100^ SMA may mediate predictive coding of an evolving familiar melody, engaging sensorimotor coupling and motor mirroring as well as auditory imagery.^39,96,101-103^ This region is likely to drive preserved melody recognition in AD^49,59^ and potentially, support an island of retained semantic competence in tvFTD.^29,35,64,67,104,105^ Involvement of IFG here is also in line with previous work on musical familiarity processing^106^: this region may have a role in predictive coding of the syntax of familiar melodies (a property that sets these apart from most other categories of sensory objects)^107-110^ as well as a more generic role in disambiguating familiar from novel sensory information.^111,112^ The processing of melodic novelty (the ‘inverse’ of familiarity) across groups here engaged postero-medial cortical components of the default mode network (posterior cingulate and precuneus) previously implicated in coding novelty in music and other sensory domains, both in the healthy brain and in AD.^47,59,62,113,114^

A major motivation for the present work was to identify syndromic functional neuroanatomical signatures of altered musical object coding in AD and tvFTD. The fMRI differences between participant groups here were relatively modest, which may speak to the intrinsic robustness of music processing mechanisms or the limited sensitivity of our paradigm to reveal such differences. Nevertheless, syndromic signatures did emerge in the higher-order processing of semantic familiarity in musical sources and source-invariant patterns, rather than more elementary source discrimination or perceptual analysis of auditory patterns. On the one hand, the reduced activation of right temporal polar cortex in the tvFTD group compared with HV during timbre familiarity processing corroborates the role of anterior temporal lobes in semantic cognition^100^ and more specific evidence for impaired recognition of musical instruments in tvFTD.^35,70^ On the other hand, the increased activation of insula in the tvFTD group may reflect the role this region plays in coding structural regularities and emotional content of music and other biologically salient, natural auditory objects (such as human voices) versus synthetic sound sources,^39,52,115,116^ modulated by pleasantness and sensorimotor coupling.^39,117,118^ These mechanisms may assume preeminence in the context of less efficient semantic differentiation of sound sources in tvFTD.

In line with this interpretation, patients with tvFTD also showed increased temporal polar activation relative to HV in processing melody constancy. Though direct evidence in the musical domain is limited, both auditory and visual apperceptive agnosias may follow anterior temporal lobe damage or degeneration,^11,119^ and top-down projections from this region play a key role in the apperceptive analysis of faces and environmental sounds.^29,120^ Moreover, aberrantly increased anterior temporal lobe activation during apperceptive processing of environmental sound categories has been demonstrated previously in tvFTD.^29^ In contrast, for processing melody familiarity, the AD group showed reduced activation of SMA compared to both the HV and tvFTD groups. While this initially may seem at odds with previous work demonstrating preserved musical semantic memory linked to SMA engagement in AD,^49,59^ the familiar melodies here were presented in a reduced non-canonical form (see Table S1), which may have imposed apperceptive processing demands even in the absence of interpolated timbre changes.

Dissonance, a music-specific property, was associated with activation of the amygdala in the HV group, consistent with previous work^37,121,122^; however, no group fMRI signatures of dissonance processing were identified for AD or tvFTD. The neuroanatomy of dissonance perception is not well established. Emerging evidence suggests that this may depend on task and contextual demands, serving to modulate other object coding mechanisms rather than constituting a distinct neural signature.^123,124^

The post-scan behavioural results align broadly with the differential activation profiles in the syndromic groups; relative to HV, patients with tvFTD had a deficit of instrument familiarity processing, while patients with AD had deficits of melody familiarity and constancy processing, aligning with previous neuropsychological evidence in these syndromes.^35,57,70,125-127^ Both on the post-scan musical tasks and on standardized neuropsychological measures (Table 1), the AD group performed less well overall than the tvFTD group, raising the possibility that the syndromic activation differences here may at least in part reflect differential disease severity. However, there was no effect of behavioural task performance on BOLD signal change in any of the participant groups. Further, the tvFTD group showed increased activation of the temporo-polar cortex versus HV, while the SMA locus of reduced activation in the AD group fell outside the zone of atrophy for this group in the VBM analysis. Taken together, these results argue that the activation alterations in the AD and tvFTD groups are not attributable simply to compensatory mechanisms or a direct consequence of regional grey matter loss. Instead, these disease-associated profiles may reflect aberrant reorganisation of the neural networks engaged in musical object processing, analogous to that described previously for environmental sound processing and musical episodic memory in these syndromes.^29,59^ This functional reorganisation potentially involves areas remote from major loci of structural neurodegeneration and heightened as well as attenuated neural activity. Our findings may have wider relevance for interpreting the neural mechanisms in AD and FTD that support the analysis of complex, real-world sensory and social environments with interacting objects and information streams, for which music serves as a model.^75,77,128,129^

This study has several limitations that should direct future work. Other important dimensions of music—especially musical emotion, reward, social cognition and autobiographical memories—warrant exploration using more naturalistic musical stimuli in analogous paradigms.^33,36,76,130^ Extension to other neurodegenerative disorders, such as Parkinson’s disease, in which music may access neurobiological and therapeutic mechanisms,^131^ is also needed. Our experimental design could be modified to incorporate an in-scanner task or for use with dynamic neuroimaging modalities such as magnetoencephalography; these would provide a more nuanced picture of the neurobiological mechanisms underpinning music processing in AD and FTD. Group sizes here were relatively small: larger study cohorts would boost statistical power to detect effects, particularly in heterogeneous disorders such as FTD.^132-134^ Perhaps the most pressing need is to establish how the functional neuroanatomy of musical object processing in AD and FTD predicts the retained capacities and specific difficulties exhibited by people with these diseases when navigating the challenging sensory and social milieu of daily life.^75,133-135^ Ultimately, defining the neural signatures of these abilities and deficits will inform the design and evaluation of personalized music-based interventions for people living with dementia, an emerging focus of considerable neurobiological and clinical interest.^136,137^

## Conclusions

In demonstrating that perceptual and semantic mechanisms of musical object analysis may be partly preserved in AD and tvFTD, our findings suggest neural substrates for the clinical impression that musical capacities can sometimes endure remarkably unscathed in dementia. However, this work has also revealed that certain computationally demanding aspects of music processing—such as the recognition of altered melodies and musical instruments—are differentially affected by these dementia syndromes, with aberrant engagement of cortical networks targeted in tvFTD. Music is a promising paradigm for exploring how neurodegenerative diseases impact the neural decoding of complex sensory environments, with a view to developing novel diagnostic and therapeutic tools.

## Supplementary Material

fcag161_Supplementary_Data

## Data Availability

The data that support the findings of this study are available on request from the corresponding author. In line with the ethics approvals governing the study, the data are not fully publicly available as they include information that could compromise the confidentiality of the research participants. Audio files of the musical stimuli and relevant scripts can be found on the Open Science Framework website here; see also Supplementary material.

## References

[1] Armstrong RA . Alzheimer's disease and the eye. J Optom. 2009;2(3):103–111.

[2] Hardy CJD, Marshall CR, Golden HL, et al Hearing and dementia. J Neurol. 2016;263(11):2339–2354. 27372450 10.1007/s00415-016-8208-y PMC5065893

[3] Hardy CJD, Taylor-Rubin C, Taylor B, et al Symptom-led staging for semantic and non-fluent/agrammatic variants of primary progressive aphasia. Alzheimer's Dement. 2024;20(1):195–210. 37548125 10.1002/alz.13415 PMC10917001

[4] Johnson JCS, Marshall CR, Weil RS, Bamiou DE, Hardy CJD, Warren JD. Hearing and dementia: From ears to brain. Brain. 2021;144(2):391–401. 33351095 10.1093/brain/awaa429 PMC7940169

[5] Levett BA, Chandra A, Jiang J, et al Hearing impairment and dementia: Cause, catalyst or consequence? J Neurol. 2025;272(6):402. 40377748 10.1007/s00415-025-13140-x PMC12084262

[6] Salobrar-Garcia E, de Hoz R, Ramirez AI, et al Changes in visual function and retinal structure in the progression of Alzheimer's disease. PLoS One. 2019;14(8):e0220535. 31415594 10.1371/journal.pone.0220535 PMC6695171

[7] Liang Y, Pertzov Y, Nicholas JM, et al Visual short-term memory binding deficit in familial Alzheimer's disease. Cortex. 2016;78:150–164. 27085491 10.1016/j.cortex.2016.01.015 PMC4865502

[8] Parra MA, Sala SD, Abrahams S, Logie RH, Méndez LG, Lopera F. Specific deficit of colour-colour short-term memory binding in sporadic and familial Alzheimer's disease. Neuropsychologia. 2011;49(7):1943–1952. 21435348 10.1016/j.neuropsychologia.2011.03.022

[9] Gaynor LS, Curiel Cid RE, Penate A, et al Visual object discrimination impairment as an early predictor of mild cognitive impairment and Alzheimer's disease. J Int Neuropsychol Soc. 2019;25(7):688–698. 31111810 10.1017/S1355617719000316 PMC6688903

[10] Warren JD, Warrington EK. Cognitive neuropsychology of dementia syndromes. In: Growdon JH, Rossor MN, eds. Blue books of neurology. Butterworth-Heinemann; 2007:329–380.

[11] Goll JC, Crutch SJ, Loo JH, et al Non-verbal sound processing in the primary progressive aphasias. Brain. 2010;133(Pt 1):272–285. 19797352 10.1093/brain/awp235 PMC2801322

[12] Ralph MAL, Patterson K. *Generalization and differentiation in semantic memory*: Insights from semantic dementia. Ann N Y Acad Sci. 2008;1124:61–76. 18400924 10.1196/annals.1440.006

[13] Greicius MD, Srivastava G, Reiss AL, Menon V. Default-mode network activity distinguishes Alzheimer's disease from healthy aging: Evidence from functional MRI. Proc Natl Acad Sci U S A. 2004;101(13):4637–4642. 15070770 10.1073/pnas.0308627101 PMC384799

[14] Supekar K, Menon V, Rubin D, Musen M, Greicius MD. Network analysis of intrinsic functional brain connectivity in Alzheimer's disease. PLoS Comput Biol. 2008;4(6):e1000100. 18584043 10.1371/journal.pcbi.1000100 PMC2435273

[15] Buckner RL, Sepulcre J, Talukdar T, et al Cortical hubs revealed by intrinsic functional connectivity: Mapping, assessment of stability, and relation to Alzheimer's disease. J Neurosci. 2009;29(6):1860–1873. 19211893 10.1523/JNEUROSCI.5062-08.2009 PMC2750039

[16] Seeley WW, Crawford RK, Zhou J, Miller BL, Greicius MD. Neurodegenerative diseases target large-scale human brain networks. Neuron. 2009;62(1):42–52. 19376066 10.1016/j.neuron.2009.03.024 PMC2691647

[17] Benhamou E, Marshall CR, Russell LL, et al The neurophysiological architecture of semantic dementia: Spectral dynamic causal modelling of a neurodegenerative proteinopathy. Sci Rep. 2020;10(1):16321. 33004840 10.1038/s41598-020-72847-1 PMC7530731

[18] Goll JC, Crutch SJ, Warren JD. Central auditory disorders: Toward a neuropsychology of auditory objects. Curr Opin Neurol. 2010;23(6):617–627. 20975559 10.1097/WCO.0b013e32834027f6 PMC3374998

[19] Griffiths TD, Warren JD. What is an auditory object? Nat Rev Neurosci. 2004;5(11):887–892. 15496866 10.1038/nrn1538

[20] Bregman AS . Auditory scene analysis: The perceptual organization of sound. MIT press; 1994.

[21] McAdams S . The perceptual representation of timbre, eds. Timbre: Acoustics, perception, and cognition. Springer; 2019:23–57.

[22] Benhamou E, Warren JD. Disorders of music processing in dementia, eds. Music and the aging brain. Elsevier Academic Press; 2020:107–149.

[23] Golden HL, Clark CN, Nicholas JM, et al Music perception in dementia. J Alzheimers Dis. 2016;55:933–949. 10.3233/JAD-160359 PMC5260961 27802226

[24] Benhamou E, Zhao S, Sivasathiaseelan H, et al Decoding expectation and surprise in dementia: The paradigm of music. Brain Commun. 2021;3(3):fcab173. 34423301 10.1093/braincomms/fcab173 PMC8376684

[25] Hailstone JC, Omar R, Henley SMD, Frost C, Kenward MG, Warren JD. It's not what you play, it's how you play it: Timbre affects perception of emotion in music. Q J Exp Psychol. 2009;62(11):2141–2155. 10.1080/17470210902765957 PMC2683716 19391047

[26] Koelsch S, Gunter T, Friederici AD, Schroger E. Brain indices of music processing: “Nonmusicians” are musical. J Cogn Neurosci. 2000;12(3):520–541. 10931776 10.1162/089892900562183

[27] Clark CN, Downey LE, Warren JD. Music biology: All this useful beauty. Curr Biol. 2014;24(6):R234–R237. 24650910 10.1016/j.cub.2014.02.013

[28] Clark CN, Warren JD. Music, memory and mechanisms in Alzheimer’s disease: Figure 1. Brain. 2015;138(8):2122–2125. 26205838 10.1093/brain/awv148 PMC4511859

[29] Goll JC, Ridgway GR, Crutch SJ, Theunissen FE, Warren JD. Nonverbal sound processing in semantic dementia: A functional MRI study. Neuroimage. 2012;61(1):170–180. 22405732 10.1016/j.neuroimage.2012.02.045 PMC3398766

[30] Turnbull OH, Carey DP, McCarthy RA. The neuropsychology of object constancy. J Int Neuropsychol Soc. 1997;3(3):288–298. 9161108

[31] Warrington EK, Taylor AM. Two categorical stages of object recognition. Perception. 1978;7(6):695–705. 740510 10.1068/p070695

[32] Webster JC, Woodhead MM, Carpenter A. Perceptual constancy in complex sound identification. Br J Psychol. 1970;61(4):481–489. 5487459 10.1111/j.2044-8295.1970.tb01267.x

[33] Fritz T, Jentschke S, Gosselin N, et al Universal recognition of three basic emotions in music. Curr Biol. 2009;19(7):573–576. 19303300 10.1016/j.cub.2009.02.058

[34] Navarro-Cáceres M, Caetano M, Bernardes G, Sánchez-Barba M, Sánchez-Jara JM. A computational model of tonal tension profile of chord progressions in the tonal interval space. Entropy (Basel). 2020;22(11):1291. 33287059 10.3390/e22111291 PMC7712964

[35] Omar R, Hailstone JC, Warren JE, Crutch SJ, Warren JD. The cognitive organization of music knowledge: A clinical analysis. Brain. 2010;133(4):1200–1213. 20142334 10.1093/brain/awp345 PMC2850578

[36] Blood AJ, Zatorre RJ, Bermudez P, Evans AC. Emotional responses to pleasant and unpleasant music correlate with activity in paralimbic brain regions. Nat Neurosci. 1999;2(4):382–387. 10204547 10.1038/7299

[37] Koelsch S, Fritz T, VC DY, Müller K, Friederici AD. Investigating emotion with music: An fMRI study. Hum Brain Mapp. 2006;27(3):239–250. 16078183 10.1002/hbm.20180 PMC6871371

[38] Pallesen KJ, Brattico E, Bailey C, et al Emotion processing of major, minor, and dissonant chords: A functional magnetic resonance imaging study. Ann N Y Acad Sci. 2005;1060:450–453. 16597801 10.1196/annals.1360.047

[39] Bellmann OT, Asano R. Neural correlates of musical timbre: An ALE meta-analysis of neuroimaging data. Front Neurosci. 2024;18:1373232. 38952924 10.3389/fnins.2024.1373232 PMC11215185

[40] Janata P . Neural basis of music perception. Handb Clin Neurol. 2015;129:187–205. 25726270 10.1016/B978-0-444-62630-1.00011-1

[41] Wei Y, Gan L, Huang X. A review of research on the neurocognition for timbre perception. Front Psychol. 2022;13:869475. 35422736 10.3389/fpsyg.2022.869475 PMC9001888

[42] Krishnan S, Lima CF, Evans S, et al Beatboxers and guitarists engage sensorimotor regions selectively when listening to the instruments they can play. Cereb Cortex. 2018;28(11):4063–4079. 30169831 10.1093/cercor/bhy208 PMC6188551

[43] Margulis EH, Mlsna LM, Uppunda AK, Parrish TB, Wong PC. Selective neurophysiologic responses to music in instrumentalists with different listening biographies. Hum Brain Mapp. 2009;30(1):267–275. 18072277 10.1002/hbm.20503 PMC6871237

[44] Ogg M, Moraczewski D, Kuchinsky SE, Slevc LR. Separable neural representations of sound sources: Speaker identity and musical timbre. Neuroimage. 2019;191:116–126. 30731247 10.1016/j.neuroimage.2019.01.075

[45] Segado M, Zatorre RJ, Penhune VB. Effector-independent brain network for auditory-motor integration: FMRI evidence from singing and cello playing. Neuroimage. 2021;237:118128. 33989814 10.1016/j.neuroimage.2021.118128

[46] Sternin A, McGarry LM, Owen AM, Grahn JA. The effect of familiarity on neural representations of music and language. J Cogn Neurosci. 2021;33(8):1595–1611. 34496377 10.1162/jocn_a_01737

[47] Agustus JL, Golden HL, Callaghan MF, et al Melody processing characterizes functional neuroanatomy in the aging brain. Front Neurosci. 2018;12:815. 30524219 10.3389/fnins.2018.00815 PMC6262413

[48] Herholz SC, Halpern AR, Zatorre RJ. Neuronal correlates of perception, imagery, and memory for familiar tunes. J Cogn Neurosci. 2012;24(6):1382–1397. 22360595 10.1162/jocn_a_00216

[49] Jacobsen J-H, Stelzer J, Fritz TH, Chételat G, La Joie R, Turner R. Why musical memory can be preserved in advanced Alzheimer’s disease. Brain. 2015;138(8):2438–2450. 26041611 10.1093/brain/awv135

[50] Peretz I, Gosselin N, Belin P, Zatorre RJ, Plailly J, Tillmann B. Music lexical networks: The cortical organization of music recognition. Ann N Y Acad Sci. 2009;1169:256–265. 19673789 10.1111/j.1749-6632.2009.04557.x

[51] Sikka R, Cuddy LL, Johnsrude IS, Vanstone AD. An fMRI comparison of neural activity associated with recognition of familiar melodies in younger and older adults. Front Neurosci. 2015;9:135025. 10.3389/fnins.2015.00356 PMC4594019 26500480

[52] Vuong V, Hewan P, Perron M, Thaut MH, Alain C. The neural bases of familiar music listening in healthy individuals: An activation likelihood estimation meta-analysis. Neurosci Biobehav Rev. 2023;154:105423. 37839672 10.1016/j.neubiorev.2023.105423

[53] Foster NE, Zatorre RJ. A role for the intraparietal sulcus in transforming musical pitch information. Cereb Cortex. 2010;20(6):1350–1359. 19789184 10.1093/cercor/bhp199

[54] Han Y-A, Hsieh P-J. Melody transposition tolerance in the human cortex: An fMRI adaptation and MVPA investigation. Imaging Neurosci (Camb). 2024;2:1–13. 10.1162/imag_a_00352 PMC12290743 40800331

[55] Lee Y-S, Janata P, Frost C, Hanke M, Granger R. Investigation of melodic contour processing in the brain using multivariate pattern-based fMRI. Neuroimage. 2011;57(1):293–300. 21315158 10.1016/j.neuroimage.2011.02.006

[56] Schindler A, Herdener M, Bartels A. Coding of melodic gestalt in human auditory cortex. Cerebral Cortex. 2013;23(12):2987–2993. 22989579 10.1093/cercor/bhs289 PMC3827712

[57] Goll JC, Kim LG, Hailstone JC, et al Auditory object cognition in dementia. Neuropsychologia. 2011;49(9):2755–2765. 21689671 10.1016/j.neuropsychologia.2011.06.004 PMC3202629

[58] Samson S, Baird A, Moussard A, Clément S. Does pathological aging affect musical learning and memory? Music Percept. 2012;29(5):493–500.

[59] Slattery CF, Agustus JL, Paterson RW, et al The functional neuroanatomy of musical memory in Alzheimer's disease. Cortex. 2019;115:357–370. 30846199 10.1016/j.cortex.2019.02.003 PMC6525150

[60] Vanstone AD, Cuddy LL. Musical memory in Alzheimer disease. Aging Neuropsychol Cogn. 2009;17(1):108–128. 10.1080/13825580903042676 19657762

[61] Downar J, Crawley AP, Mikulis DJ, Davis KD. A cortical network sensitive to stimulus salience in a neutral behavioral context across multiple sensory modalities. J Neurophysiol. 2002;87(1):615–620. 11784775 10.1152/jn.00636.2001

[62] Herdener M, Esposito F, di Salle F, et al Musical training induces functional plasticity in human hippocampus. J Neurosci. 2010;30(4):1377–1384. 20107063 10.1523/JNEUROSCI.4513-09.2010 PMC3842475

[63] Hunkin NM, Mayes AR, Gregory LJ, et al Novelty-related activation within the medial temporal lobes. Neuropsychologia. 2002;40(8):1456–1464. 11931949 10.1016/s0028-3932(01)00200-7

[64] Hailstone JC, Omar R, Warren JD. Relatively preserved knowledge of music in semantic dementia. J Neurol Neurosurg Psychiatry. 2009;80(7):808–809. 19531690 10.1136/jnnp.2008.153130 PMC3775126

[65] Hsieh S, Hornberger M, Piguet O, Hodges JR. Neural basis of music knowledge: Evidence from the dementias. Brain. 2011;134(9):2523–2534. 21857031 10.1093/brain/awr190

[66] Johnson JK, Chang CC, Brambati SM, et al Music recognition in frontotemporal lobar degeneration and Alzheimer disease. Cogn Behav Neurol. 2011;24(2):74–84. 21617528 10.1097/WNN.0b013e31821de326 PMC3691095

[67] Weinstein J, Koenig P, Gunawardena D, McMillan C, Bonner M, Grossman M. Preserved musical semantic memory in semantic dementia. Arch Neurol. 2011;68(2):248–250. 21320991 10.1001/archneurol.2010.364 PMC3268521

[68] Grube M, Bruffaerts R, Schaeverbeke J, et al Core auditory processing deficits in primary progressive aphasia. Brain. 2016;139(6):1817–1829. 27060523 10.1093/brain/aww067 PMC4892752

[69] Halpern AR, Golden HL, Magdalinou N, Witoonpanich P, Warren JD. Musical tasks targeting preserved and impaired functions in two dementias. Ann N Y Acad Sci. 2015;1337(1):241–248. 25773640 10.1111/nyas.12616 PMC4401999

[70] Hailstone JC, Crutch SJ, Vestergaard MD, Patterson RD, Warren JD. Progressive associative phonagnosia: A neuropsychological analysis. Neuropsychologia. 2010;48(4):1104–1114. 20006628 10.1016/j.neuropsychologia.2009.12.011 PMC2833414

[71] Samson S, Zatorre RJ, Ramsay JO. Deficits of musical timbre perception after unilateral temporal-lobe lesion revealed with multidimensional scaling. Brain. 2002;125(3):511–523. 11872609 10.1093/brain/awf051

[72] Samson S, Zatorre RJ. Contribution of the right temporal lobe to musical timbre discrimination. Neuropsychologia. 1994;32(2):231–240. 8190246 10.1016/0028-3932(94)90008-6

[73] Downey LE, Blezat A, Nicholas J, et al Mentalising music in frontotemporal dementia. Cortex. 2013;49(7):1844–1855. 23107380 10.1016/j.cortex.2012.09.011 PMC3701324

[74] Hsieh S, Hornberger M, Piguet O, Hodges JR. Brain correlates of musical and facial emotion recognition: Evidence from the dementias. Neuropsychologia. 2012;50(8):1814–1822. 22579645 10.1016/j.neuropsychologia.2012.04.006

[75] van't Hooft JJ, Hartog WL, Braun M, et al Musicality and social cognition in dementia: Clinical and anatomical associations. Brain Commun. 2024;6(6):fcae429. 39678365 10.1093/braincomms/fcae429 PMC11642622

[76] Agustus JL, Mahoney CJ, Downey LE, et al Functional MRI of music emotion processing in frontotemporal dementia. Ann N Y Acad Sci. 2015;1337(1):232–240. 25773639 10.1111/nyas.12620 PMC4402026

[77] van't Hooft JJ, Fieldhouse JLP, Singleton EH, et al Music appreciation phenotypes in patients with frontotemporal dementia: A pilot study. Int J Geriatr Psychiatry. 2022;37(9):1-9. 10.1002/gps.5793 PMC9544804 35962477

[78] Omar R, Henley SM, Bartlett JW, et al The structural neuroanatomy of music emotion recognition: Evidence from frontotemporal lobar degeneration. Neuroimage. 2011;56(3):1814–1821. 21385617 10.1016/j.neuroimage.2011.03.002 PMC3092986

[79] Belder CRS, Chokesuwattanaskul A, Marshall CR, Hardy CJD, Rohrer JD, Warren JD. The problematic syndrome of right temporal lobe atrophy: Unweaving the phenotypic rainbow. Front Neurol. 2023;13:1082828. 36698890 10.3389/fneur.2022.1082828 PMC9868162

[80] Ulugut H, Bertoux M, Younes K, et al Clinical recognition of frontotemporal dementia with right anterior temporal predominance: A multicenter retrospective cohort study. Alzheimers Dement. 2024;20(8):5647–5661. 38982845 10.1002/alz.14076 PMC11350044

[81] Younes K, Borghesani V, Montembeault M, et al Right temporal degeneration and socioemotional semantics: Semantic behavioural variant frontotemporal dementia. Brain. 2022;145(11):4080–4096. 35731122 10.1093/brain/awac217 PMC10200288

[82] Dubois B, Feldman HH, Jacova C, et al Advancing research diagnostic criteria for Alzheimer's disease: The IWG-2 criteria. Lancet Neurol. 2014;13(6):614–629. 24849862 10.1016/S1474-4422(14)70090-0

[83] Gorno-Tempini ML, Hillis AE, Weintraub S, et al Classification of primary progressive aphasia and its variants. Neurology. 2011;76(11):1006–1014. 21325651 10.1212/WNL.0b013e31821103e6 PMC3059138

[84] Rascovsky K, Hodges JR, Knopman D, et al Sensitivity of revised diagnostic criteria for the behavioural variant of frontotemporal dementia. Brain. 2011;134(Pt 9):2456–2477. 21810890 10.1093/brain/awr179 PMC3170532

[85] Hall DA, Haggard MP, Akeroyd MA, et al Sparse” temporal sampling in auditory fMRI. Hum Brain Mapp. 1999;7(3):213–223. 10194620 10.1002/(SICI)1097-0193(1999)7:3<213::AID-HBM5>3.0.CO;2-N PMC6873323

[86] Desikan RS, Segonne F, Fischl B, et al An automated labeling system for subdividing the human cerebral cortex on MRI scans into gyral based regions of interest. Neuroimage. 2006;31(3):968–980. 16530430 10.1016/j.neuroimage.2006.01.021

[87] Jenkinson M, Beckmann CF, Behrens TE, Woolrich MW, Smith SM. Fsl. Neuroimage. 2012;62(2):782–790. 21979382 10.1016/j.neuroimage.2011.09.015

[88] Gaetano J . Signal detection theory calculator [Excel Workbook] Version 1.2. 2017.

[89] Kloke JD, McKean JW. Rfit: Rank-based estimation for linear models. R J. 2012;4(2):57–64.

[90] Mole JA, Baker IW, Munoz O, et al Avian agnosia: A window into auditory semantics. Neuropsychologia. 2019;134:107219. 31593713 10.1016/j.neuropsychologia.2019.107219 PMC6891886

[91] Muhammed L, Hardy CJD, Russell LL, et al Agnosia for bird calls. Neuropsychologia. 2018;113:61–67. 29572063 10.1016/j.neuropsychologia.2018.03.024 PMC5946901

[92] Griffiths TD, Warren JD. The planum temporale as a computational hub. Trends Neurosci. 2002;25(7):348–353. 12079762 10.1016/s0166-2236(02)02191-4

[93] Kumar S, Stephan KE, Warren JD, Friston KJ, Griffiths TD. Hierarchical processing of auditory objects in humans. PLoS Comput Biol. 2007;3(6):e100. 17542641 10.1371/journal.pcbi.0030100 PMC1885275

[94] Warren JD, Jennings AR, Griffiths TD. Analysis of the spectral envelope of sounds by the human brain. Neuroimage. 2005;24(4):1052–1057. 15670682 10.1016/j.neuroimage.2004.10.031

[95] von Kriegstein K, Warren JD, Ives DT, Patterson RD, Griffiths TD. Processing the acoustic effect of size in speech sounds. Neuroimage. 2006;32(1):368–375. 16644240 10.1016/j.neuroimage.2006.02.045

[96] Halpern AR, Zatorre RJ, Bouffard M, Johnson JA. Behavioral and neural correlates of perceived and imagined musical timbre. Neuropsychologia. 2004;42(9):1281–1292. 15178179 10.1016/j.neuropsychologia.2003.12.017

[97] Patterson RD, Uppenkamp S, Johnsrude IS, Griffiths TD. The processing of temporal pitch and melody information in auditory Cortex. Neuron. 2002;36(4):767–776. 12441063 10.1016/s0896-6273(02)01060-7

[98] Warren JD, Uppenkamp S, Patterson RD, Griffiths TD. Separating pitch chroma and pitch height in the human brain. Proc Natl Acad Sci U S A. 2003;100(17):10038–10042. 12909719 10.1073/pnas.1730682100 PMC187755

[99] Pereira CS, Teixeira J, Figueiredo P, Xavier J, Castro SL, Brattico E. Music and emotions in the brain: Familiarity matters. PLoS One. 2011;6(11):e27241. 22110619 10.1371/journal.pone.0027241 PMC3217963

[100] Ralph MAL, Jefferies E, Patterson K, Rogers TT. The neural and computational bases of semantic cognition. Nat Rev Neurosci. 2017;18(1):42–55. 27881854 10.1038/nrn.2016.150

[101] Janata P, Tomic ST, Haberman JM. Sensorimotor coupling in music and the psychology of the groove. J Exp Psychol Gen. 2012;141(1):54–75. 21767048 10.1037/a0024208

[102] Gordon CL, Cobb PR, Balasubramaniam R. Recruitment of the motor system during music listening: An ALE meta-analysis of fMRI data. PLoS One. 2018;13(11):e0207213. 30452442 10.1371/journal.pone.0207213 PMC6242316

[103] Matthews TE, Witek MAG, Lund T, Vuust P, Penhune VB. The sensation of groove engages motor and reward networks. Neuroimage. 2020;214:116768. 32217163 10.1016/j.neuroimage.2020.116768

[104] Tirigay R, Moltrasio J, Rubinstein W. Dissociations between musical semantic memory and verbal memory in a patient with behavioral variant frontotemporal dementia. Appl Neuropsychol Adult. 2025;32(1):75–84. 36416413 10.1080/23279095.2022.2148105

[105] Hodges JR, Patterson K. Semantic dementia: A unique clinicopathological syndrome. Lancet Neurol. 2007;6(11):1004–1014. 17945154 10.1016/S1474-4422(07)70266-1

[106] Platel H, Price C, Baron J-C, et al The structural components of music perception. A functional anatomical study. Brain. 1997;120(2):229–243. 9117371 10.1093/brain/120.2.229

[107] Maess B, Koelsch S, Gunter TC, Friederici AD. Musical syntax is processed in broca's area: An MEG study. Nat Neurosci. 2001;4(5):540–545. 11319564 10.1038/87502

[108] Patel AD . Language, music, syntax and the brain. Nat Neurosci. 2003;6(7):674–681. 12830158 10.1038/nn1082

[109] Tillmann B, Janata P, Bharucha JJ. Activation of the inferior frontal cortex in musical priming. Brain Res Cogn Brain Res. 2003;16(2):145–161. 12668222 10.1016/s0926-6410(02)00245-8

[110] Siman-Tov T, Granot RY, Shany O, Singer N, Hendler T, Gordon CR. Is there a prediction network? Meta-analytic evidence for a cortical-subcortical network likely subserving prediction. Neurosci Biobehav Rev. 2019;105:262–275. 31437478 10.1016/j.neubiorev.2019.08.012

[111] Downar J, Crawley AP, Mikulis DJ, Davis KD. A cortical network for the detection of novel events across multiple sensory modalities. Neuroimage. 2001;13(6):310.

[112] Greene CM, Vidaki K, Soto D. Fractionating the neural substrates of incidental recognition memory. Learn Mem. 2015;22(1):24–30. 10.1101/lm.036327.114 PMC4274326 25512574

[113] Demorest SM, Morrison SJ, Stambaugh LA, Beken M, Richards TL, Johnson C. An fMRI investigation of the cultural specificity of music memory. Soc Cogn Affect Neurosci. 2010;5(2–3):282–291. 20035018 10.1093/scan/nsp048 PMC2894677

[114] Billette OV, Ziegler G, Aruci M, et al Novelty-related fMRI responses of precuneus and medial temporal regions in individuals at risk for Alzheimer disease. Neurology. 2022;99(8):e775-e788. 35995589 10.1212/WNL.0000000000200667 PMC9484732

[115] Asano R, Lo V, Brown S. The neural basis of tonal processing in music: An ALE meta-analysis. Music Sci (Lond). 2022;5:20592043221109958.

[116] Bestelmeyer PE, Mühl C. Neural dissociation of the acoustic and cognitive representation of voice identity. NeuroImage. 2022;263:119647. 36162634 10.1016/j.neuroimage.2022.119647

[117] Uddin LQ, Nomi JS, Hebert-Seropian B, Ghaziri J, Boucher O. Structure and function of the human Insula. J Clin Neurophysiol. 2017;34(4):300–306. 28644199 10.1097/WNP.0000000000000377 PMC6032992

[118] van den Bosch I, Salimpoor VN, Zatorre RJ. Familiarity mediates the relationship between emotional arousal and pleasure during music listening. Front Hum Neurosci. 2013;7:534. 24046738 10.3389/fnhum.2013.00534 PMC3763198

[119] Busigny T, Van Belle G, Jemel B, Hosein A, Joubert S, Rossion B. Face-specific impairment in holistic perception following focal lesion of the right anterior temporal lobe. Neuropsychologia. 2014;56:312–333. 24503392 10.1016/j.neuropsychologia.2014.01.018

[120] Rapcsak SZ . Face recognition. Curr Neurol Neurosci Rep. 2019;19:1–9. 31144153 10.1007/s11910-019-0960-9

[121] Omigie D, Dellacherie D, Hasboun D, et al Intracranial markers of emotional valence processing and judgments in music. Cogn Neurosci. 2015;6(1):16–23. 25496511 10.1080/17588928.2014.988131

[122] González-García N, González MA, Rendón PL. Neural activity related to discrimination and vocal production of consonant and dissonant musical intervals. Brain Res. 2016;1643:59–69. 27134038 10.1016/j.brainres.2016.04.065

[123] Bravo F, Glogowski J, Stamatakis EA, Herfert K. Dissonant music engages early visual processing. Proc Natl Acad Sci U S A. 2024;121(30):e2320378121. 39008675 10.1073/pnas.2320378121 PMC11287129

[124] Gold BP, Mas-Herrero E, Zeighami Y, Benovoy M, Dagher A, Zatorre RJ. Musical reward prediction errors engage the nucleus accumbens and motivate learning. Proc Natl Acad Sci U S A. 2019;116(8):3310–3315. 30728301 10.1073/pnas.1809855116 PMC6386687

[125] Sugimoto A, Midorikawa A, Koyama S, Futamura A, Hieda S, Kawamura M. Picture agnosia as a characteristic of posterior cortical atrophy. Eur Neurol. 2012;68(1):34–41. 22710605 10.1159/000335589

[126] Tippett LJ, Blackwood K, Farah MJ. Visual object and face processing in mild-to-moderate Alzheimer’s disease: From segmentation to imagination. Neuropsychologia. 2003;41(4):453–468. 12559162 10.1016/s0028-3932(02)00140-9

[127] Uhlhaas PJ, Pantel J, Lanfermann H, et al Visual perceptual organization deficits in Alzheimer's dementia. Dement Geriatr Cogn Disord. 2008;25(5):465–475. 18408365 10.1159/000125671

[128] Sievers B, Parkinson C, Kohler PJ, Hughes JM, Fogelson SV, Wheatley T. Visual and auditory brain areas share a representational structure that supports emotion perception. Curr Biol. 2021;31(23):5192–5203.e4. 34644547 10.1016/j.cub.2021.09.043

[129] van't Hooft JJ, Pijnenburg YA, Sikkes SA, et al Frontotemporal dementia, music perception and social cognition share neurobiological circuits: A meta-analysis. Brain Cogn. 2021;148:105660. 33421942 10.1016/j.bandc.2020.105660

[130] Blood AJ, Zatorre RJ. Intensely pleasurable responses to music correlate with activity in brain regions implicated in reward and emotion. Proc Natl Acad Sci U S A. 2001;98(20):11818–11823. 11573015 10.1073/pnas.191355898 PMC58814

[131] Huang C-W, Tsai H-Y, Lin Y-H, Lin W-W, Lin C-H, Tseng M-T. Striatal-cortical dysconnectivity underlies somatosensory deficits in Parkinson's disease: Insights from rhythmic auditory-motor training. Neurobiol Dis. 2025;204:106778. 39719198 10.1016/j.nbd.2024.106778

[132] Belder CRS, Marshall CR, Jiang J, et al Primary progressive aphasia: Six questions in search of an answer. J Neurol. 2024;271(2):1028–1046. 37906327 10.1007/s00415-023-12030-4 PMC10827918

[133] Miller B, Yoon SJ. Frontotemporal dementia. In: Husain M, Schott JM, eds. Oxford Textbook of cognitive neurology and dementia. Oxford University Press; 2016:391-398

[134] Sivasathiaseelan H, Marshall CR, Agustus JL, et al Frontotemporal dementia: A clinical review. Semin Neurol. 2019;39(2):251–263. 30925617 10.1055/s-0039-1683379

[135] van’t Hooft JJ, Benhamou E, Herreros CA, et al Musical experience influences socio-emotional functioning in behavioural variant frontotemporal dementia. Brief research report. Front Neurol. 2024;15:1341661. 38333611 10.3389/fneur.2024.1341661 PMC10851745

[136] Spinosa V, Vitulli A, Logroscino G, Brattico E. A review on music interventions for frontotemporal aphasia and a proposal for alternative treatments. Biomedicines. 2023;11(1):84. 10.3390/biomedicines11010084 PMC9855720 36672592

[137] Sun L, Wang Q, Ai J. The underlying roles and neurobiological mechanisms of music-based intervention in Alzheimer's disease: A comprehensive review. Ageing Res Rev. 2024;96:102265. 38479478 10.1016/j.arr.2024.102265

