# Supplementary material for: Functional neuroanatomy of musical object processing in Alzheimer’s disease and frontotemporal dementia
Source: Brain Commun. 2026 May 5;8(3):fcag161. doi: 10.1093/braincomms/fcag161 (PMC13178109; doi:10.1093/braincomms/fcag161)
Supplement: fcag161_Supplementary_Data [file fcag161_supplementary_data.zip › Core_Supplementary_material.docx]

**Supplementary Material. Functional neuroanatomy of musical object processing in Alzheimer’s disease and frontotemporal dementia,**

**by LB Core et al.**

**Musical melody selection**

The familiar melodies used here were selected from a previously collected in-house dataset. Sixty-nine healthy older British adults between the ages of 50-78 years (mean = 59.4 years, standard deviation = 8.0 years) heard excerpts of famous melodies and pseudo-reversed versions played using a piano timbre. They rated the familiarity (1 = not familiar, 2 = slightly familiar, 3 = somewhat familiar, 4 = moderately familiar, 5 = extremely familiar) and emotional valence (1 = very sad, 2 = sad, 3 = neutral, 4 = happy, 5 = very happy) of each melody.

Thirty-four melodies each with mean familiarity rating ≥ 3.5 were used in the functional MRI experiment. We ensured that the stimulus set contained a range of emotional valence ratings. Melodies with native keys (e.g., pop songs) were kept in their original key signatures; a wide range of key signatures was represented in the final stimulus set. The healthy volunteer group in the current study also rated the emotional valence (using the same scale) of each stimulus in the familiar melody and unfamiliar melody conditions. Characteristics of all familiar melodies used are summarised in Table S1.

In the pseudo-reversed versions of the melodies, the pitch sequence was reversed (with some additional minor modifications, see main text) but the rhythmic structure was the same so that the tightest control stimulus possible was used. Previous work has indicated that pitch interval cues are more relevant for melody recognition than rhythmic cues.^1^

The piano and harp timbres used here represented naturalistic timbres although they were technically synthesised, as they were generated from MuseScore®4 software. However, they were still categorised as ‘natural’ in this experiment as they were intentionally constructed to mimic the sound of the corresponding instruments. The researchers carefully selected these instrument timbres from MuseScore®4’s large library of timbres because they sounded genuine; piloting sessions with healthy volunteers further confirmed this choice, and the healthy volunteers in the current experiment performed at ceiling when asked to discriminate the natural from artificial instrument timbres in the instrument familiarity task (Table S8, Figure 5).

**Background neuropsychological assessments**

We used abbreviated or adapted versions of some general neuropsychological tests shown in Table 1 to reduce participant fatigue. We used the Boston Naming Test 30-item even version^2^ and implemented the short form administration procedure for the Judgment of Line Orientation.^3^

The pitch change detection test was a bespoke, non-standardised test we have developed previously.^4^ Participants heard a series of 20 pure tones of fixed duration (2 seconds), synthesised in MATLAB®. The centre pitch ranged from 230-270 Hertz (Hz), and the absolute intensity was varied. Ten tones were constant in pitch, while the remaining tones had linearly ascending pitch; for ascending tones, the rate of pitch change was 0.3-0.4 octaves per second. Tones with fixed and changing pitch were presented in a randomised order. On each trial, the task was to decide if the tone changed or stayed the same.

**Peripheral hearing assessment: pure tone audiometry procedure**

To assess peripheral hearing function, each participant underwent pure tone audiometry following British Society of Audiology guidelines.^5^ Participants heard tones in each ear at 250, 500, 1000, 2000, 4000, and 8000 Hz. A staircase procedure was used to determine the minimum threshold measured in decibels for each frequency and ear. The average minimum threshold across the frequencies in the 250-4000 Hz range was computed for each ear, and the better ear average is reported here. We assessed this extended frequency range (500-4000 Hz is typically used in speech research^6^) because some of the notes in the musical stimuli went below 500 Hz.

**Quality control of neuroimaging data**

All structural and functional neuroimaging data were visually inspected for quality control, leading to the exclusion of nine participants. For two participants, this was due to incidental findings on their structural scans. For the remaining seven participants, this was due to excessive movement during the functional scans exceeding 6 mm or 6 degrees in any direction (this lenient criterion was used due to the rarity of potential participants with a diagnosis of rtvFTD).

Of the participants included in analyses, usable data were only available for the first three fMRI trial runs for three people. Eight trials across four participants were of poor quality; this was accounted for in the first-level analyses for those participants by including a covariate for low-quality trials since individual trials cannot be removed from the dataset without disrupting the timeseries.

**Structural neuroanatomical data pre-processing and analysis procedures**

The T1-weighted structural scans were manually reoriented to the anterior commissure and segmented into grey matter, white matter, and cerebrospinal fluid to create a study-specific template using the Shoot Toolbox. The grey matter images were normalised to this template and transformed to Montreal Neurological Institute space. Smoothing was applied to the images using a Gaussian kernel with full width half maximum of 6 mm. We used a customised explicit brain mask derived by maximising the correlation between the binary mask and the average of the images to avoid potential voxel dropout due to local regional atrophy using the Masking Toolbox.^7^ We compared grey matter volume for each patient group versus the healthy volunteer group using two-sample t-tests, with mean-centred covariates of age and total intracranial volume. We used voxel-wise statistics and report peak voxels thresholded at a family-wise error rate of *P* < 0.05 across the whole brain to correct for multiple voxel-wise comparisons. We created a mean structural brain image based on normalised T1-weighted images from all participants to overlay patterns of atrophy and functional activations.

**Effect of behavioural task performance on brain activation**

We conducted additional fMRI analyses to examine if performance on the behavioural post-scan music tasks influenced brain activation for the analyses where we observed significant differences between either patient group compared to healthy volunteers (see Table 2). Thus, these additional models were conducted for the melody familiarity, melody constancy, and instrument familiarity contrasts (but not the timbre change, melody novelty, or dissonance contrasts). The instrument familiarity and melody constancy models contained fewer participants due to missing behavioural data (see Table S8). In the second-level group analysis, we included performance (sensitivity, A-prime) on the relevant post-scan behavioural task and its interaction with group as an additional covariate to age and scanner protocol.

There was no main effect of behaviour nor any group by behaviour interaction, in any of our pre-specified anatomical regions of interest, using a threshold of *P* < 0.05 after family-wise error correction for multiple voxel-wise comparisons.

**Stimulus audio files and scripts**

Audio files of the musical stimuli and relevant scripts can be found on the Open Science Framework website [here](https://osf.io/azcf7/overview?view_only=12925453e1234bc9b4f3c512f5fc1b54).

The audio files are organised by experimental condition but were presented in a fixed pseudo-randomised order during fMRI scanning. The following scripts are included:

- 1_RootMeanSquare.m 🡪 MATLAB® script for fixing the root mean square level of the audio files
- 2_fMRIStimulusPresentation.m 🡪 MATLAB® script used for stimulus presentation during fMRI scanning
- 3_StimulusCharacteristics 🡪 R script used to compare characteristics across stimuli of differing timbres (Table S3)
- 4_DemNpsych.R 🡪 R script used to assess participant group differences in demographic, clinical, and neuropsychological characteristics (Table 1)
- 5_PostscanTasks.R 🡪 R script used to assess participant group differences in behavioural post-scan musical task performance (Table S8, Figure 5)

**Table S1. Characteristics of familiar melodies used in the experiment**

| **Melody** | **Genre** | **Lyrics** | **Timbre** | **Key** | **Tempo**  (bpm) | **Fam** | **Valence** |
| --- | --- | --- | --- | --- | --- | --- | --- |
| Blue Danube (Strauss) | Classical | N | Synth B | D Maj | 176 | 3.9 | 4.0 |
| Boléro (Ravel) | Classical | N | Harp | C Maj | 72 | 4.0 | 3.4 |
| Dance of the Sugar Plum Fairy (Tchaikovsky) | Classical | N | Piano | E Min | 120 | 3.9 | 3.6 |
| Do Re Mi (Rodgers) | Musical | Y | Harp | Bb Maj | 120 | 4.4 | 4.1 |
| Eine Kleine Nachtmusik (Mozart) | Classical | N | Synth A | G Maj | 120 | 4.0 | 4.3 |
| For He's a Jolly Good Fellow (Uncertain) | Trad | Y | Synth A | F Maj | 140 | 4.7 | 4.3 |
| Für Elise (Beethoven) | Classical | N | Synth B | A Min | 66 | 4.2 | 4.0 |
| Habanera from Carmen (Bizet) | Classical | Y | Harp | D Min | 90 | 3.7 | 3.5 |
| Hallelujah Chorus (Handel) | Classical | Y | Synth A | G Maj | 120 | 3.5 | 3.9 |
| Happy Birthday (Uncertain) | Trad | Y | Piano | A Maj | 100 | 4.9 | 4.0 |
| Hark! The Herald Angels (Mendelssohn) | Christmas | Y | Piano | G Maj | 120 | 4.5 | 3.8 |
| In the Hall of the Mountain King (Grieg) | Classical | N | Piano | D Min | 120 | 4.1 | 3.4 |
| Jingle Bells (Pierpont) | Christmas | Y | Harp | Bb Maj | 120 | 4.9 | 4.3 |
| Jupiter, from The Planets (Holst) | Classical | Y^a^ | Synth B | C Min | 88 | 3.8 | 3.5 |
| London Bridge is Falling Down (Uncertain) | Nursery | Y | Synth A | B Maj | 120 | 4.4 | 3.7 |
| Lullaby (Brahms) | Classical | Y | Synth A | Eb Maj | 100 | 3.8 | 3.3 |
| Memory (Lloyd Webber) | Musical | Y | Synth B | Eb Maj | 100 | 4.1 | 2.7 |
| Ode to Joy (Beethoven) | Classical | Y | Synth B | D Maj | 120 | 3.6 | 3.8 |
| Ride of the Valkyries (Wagner) | Classical | N | Harp | B Min | 160 | 3.5 | 4.1 |
| Row, Row, Row your Boat (Uncertain) | Nursery | Y | Piano | G Maj | 170 | 4.6 | 4.1 |
| Rudolph the Red Nosed Reindeer (Marks) | Christmas | Y | Synth A | G Maj | 120 | 4.7 | 3.9 |
| Singin' in the Rain (Brown) | Musical | Y | Piano | F Maj | 128 | 4.4 | 4.3 |
| Sound of Silence (Simon) | Pop | Y | Piano | F# Maj | 107 | 4.0 | 2.4 |
| Symphony No. 5, Allegro (Beethoven) | Classical | N | Harp | C Min | 156 | 3.9 | 3.4 |
| Symphony No. 9, Largo (Dvořák) | Classical | N | Piano | Ab Maj | 50 | 3.5 | 2.3 |
| Swan Lake, Theme (Tchaikovsky) | Classical | N | Synth A | E Min | 120 | 3.6 | 3.0 |
| Toccata and Fugue in D Minor (Bach) | Classical | N | Synth A | D Min | 120 | 3.9 | 3.2 |
| Twelve Days of Christmas (Uncertain) | Christmas | Y | Synth B | F Maj | 120 | 4.7 | 4.2 |
| Twinkle Twinkle Little Star (Uncertain) | Nursery | Y | Synth B | F Maj | 120 | 4.6 | 3.9 |
| Wedding March (Mendelssohn) | Classical | N | Harp | C Maj | 120 | 4.3 | 3.8 |
| When the Saints Go Marching In (Uncertain) | Trad | Y | Harp | Bb Maj | 120 | 4.4 | 4.0 |
| Winter Wonderland (Bernard) | Christmas | Y | Harp | C Maj | 120 | 4.4 | 4.1 |
| Yesterday (McCartney) | Pop | Y | Synth A | F Maj | 110 | 4.2 | 2.5 |
| You are my Sunshine (Uncertain) | Trad | Y | Synth B | Eb Maj | 126 | 4.3 | 4.3 |

Melodies are listed alphabetically by the title of the source piece from which they were excerpted (composer in parentheses); presentation order was pseudo-randomised in the experiment. All tempo values are based on the quarter note. The values for familiarity indicate the mean ratings from the previously collected in-house dataset. The values for (emotional) valence are the mean ratings from the healthy volunteer group in the current study. Eight melodies presented in the canonical form (FM condition) had piano or Synth A timbres; nine melodies had harp or Synth B timbres. ‘Lyrics’ indicates whether the source piece has associated lyrics (although no lyrics were presented in this experiment). ^a^Although Holst’s original orchestral composition did not include lyrics, most British adults also know this melody as the hymn, *I Vow to Thee my Country*. bpm, beats per minute; Fam, familiarity; FM, familiar melody; Maj, major; Min, minor; N, no; Synth, synthesiser; Trad, traditional; Y, yes.

**Table S2. Matrix of timbres used in experimental conditions**

| **Familiar melody** | **Unfamiliar melody** | **Familiar melody**  **with timbre changes** | **Unfamiliar melody**  **with timbre changes** |
| --- | --- | --- | --- |
| Piano | Synth A | PHPH | ABAB |
| Harp | Synth B | HPHP | BABA |
| Synth A | Piano | ABAB | PHPH |
| Synth B | Harp | BABA | HPHP |

The matrix indicates the timbral carriers used in the different melody conditions of the functional MRI experiment. Synth A signifies the MuseScore®4 ‘square’ synthesiser and Synth B, the ‘brightness’ synthesiser. PHPH indicates that the order of timbres used for notes within a trial was piano, harp, piano, harp; HPHP indicates the reverse ordering. ABAB indicates that the order of the timbres was Synth A, Synth B, Synth A, Synth B; BABA indicates the reverse ordering. All timbres and timbre combinations were equally represented across the experiment, and the associated melody trials were balanced for general musical characteristics (see Table S3).

**Table S3. Stimulus characteristics of the familiar melody trials employing each carrier timbre**

| **Timbre** | **Tempo**  (bpm) | **Familiarity** | **Emotional valence** |
| --- | --- | --- | --- |
| Piano | 114.4 (33.3) | 4.2 (0.4) | 3.5 (0.8) |
| Harp | 119.8 (27.6) | 4.2 (0.4) | 3.9 (0.3) |
| Synth A | 118.9 (10.5) | 4.1 (0.4) | 3.6 (0.6) |
| Synth B | 114.5 (32.2) | 4.2 (0.4) | 3.8 (0.5) |

Mean (standard deviation) values are shown; melody familiarity and emotional valence were rated on 5-point Likert scales. Familiarity ratings were derived from the previously collected in-house dataset; emotional valence ratings were provided by the healthy volunteer group in the main study. General musical characteristics were very similar among the different timbre trial subsets: there were no significant differences between timbres in tempo (Kruskal-Wallis: *X^2^*(3) = 0.22, *P* = 0.974), familiarity (ANOVA: *F*(3, 30) = 0.173, *P* = 0.914) or emotional valence (ANOVA: *F*(3, 30) = 0.827, *P* = 0.490). bpm, beats per minute; Synth, synthetic instrument.

**Table S4. Regional grey matter atrophy profiles in each patient group**

| **Group** | **Region** | **Side** | **Cluster size**  (voxels) | **Peak coordinates**  (mm) | **Z-score** | **P_FWE_-value** |
| --- | --- | --- | --- | --- | --- | --- |
| AD | Hippocampus | L | 4621 | -27, -10, -14 | 6.47 | < 0.001 |
|  | Amygdala | L |  | -21, -4, -13 | 6.04 | < 0.001 |
|  | Middle temporal gyrus | L | 93 | -63, -48, -13 | 5.13 | 0.006 |
|  | Orbitofrontal cortex | L | 207 | -9, -5, -11 | 5.10 | 0.007 |
|  | Anterior insula | L | 72 | -34, 23, -10 | 5.08 | 0.008 |
|  | Entorhinal cortex | L | 100 | -38, -12, -31 | 5.07 | 0.008 |
|  | Fornix | R | 154 | 3, -7, 7 | 5.03 | 0.010 |
| tvFTD | Entorhinal cortex | L | 41058 | -29, -10, -37 | > 8.00 | < 0.001 |
|  | Temporal pole | L |  | -32, 13, -32 | > 8.00 | < 0.001 |
|  | Anterior superior temporal gyrus | L |  | -42, 1, -21 | 7.81 | < 0.001 |
|  | Entorhinal cortex | R | 12025 | 29, -10, -37 | 6.27 | < 0.001 |
|  | Temporal pole | R |  | 40, 8, -31 | 6.26 | < 0.001 |
|  | Fusiform gyrus | R |  | 44, -13, -37 | 6.16 | < 0.001 |
|  | Insula | L | 92 | -38, -23, 11 | 5.59 | 0.001 |
|  | Supplementary motor area | R | 51 | 9, -9, 56 | 5.29 | 0.003 |
|  | Parahippocampal gyrus | R | 107 | 26, -31, -18 | 5.15 | 0.006 |
|  | Middle temporal gyrus | R | 77 | 59, -22, -7 | 5.12 | 0.007 |

The table lists areas of significantly reduced grey matter in the voxel-based morphometry analysis for each patient group compared to the healthy volunteer group thresholded at *P* < 0.05 after family-wise error correction for multiple voxel-wise comparisons over the whole brain (see also Figure S3). Only clusters of size > 50 voxels are shown due to a large number of peak voxels observed. Peak atrophy coordinates are in Montreal Neurological Institute standard space. AD, patient group with Alzheimer’s disease; FWE, family-wise error; L, left; R right; tvFTD, patient group with temporal variant frontotemporal dementia.

**Table S5. Pooled effects across groups for principal contrasts assessing musical object processing**

| **Analysis** | **Regions** | **Side** | **Cluster size**  (voxels) | **Peak coordinates** (mm) | **Z-score** |
| --- | --- | --- | --- | --- | --- |
| Timbre change | Superior temporal gyrus | R | 55 | 66, -16, 6 | 5.34 |
|  | Middle temporal gyrus | R | 121 | 64, -38, 4 | 5.19 |
|  | Middle temporal gyrus | L | 79 | -66, -40, 4 | 4.66 |
| Melody familiarity | Supplementary motor area | R | 724 | 2, 2, 64 | 6.37 |
|  | Temporal pole | L | 1290 | -52, 12, -4 | 5.71 |
|  | Precentral gyrus | L | 252 | -52, -6, 48 | 5.68 |
|  | Insula | R | 179 | 48, 6, -4 | 5.35 |
|  | Superior temporal gyrus | L | 462 | -50, -40, 18 | 5.09 |
|  | Superior temporal gyrus | R | 219 | 66, -34, 18 | 5.07 |
|  | Middle frontal gyrus | R | 93 | 44, 14, 16 | 4.91 |
|  | Inferior frontal gyrus | R | 163 | 56, 38, 2 | 4.76 |
|  | Precentral gyrus | R | 116 | 52, -6, 50 | 4.60 |
| Melody constancy | Superior temporal gyrus | L | 12 | -52, -12, 2 | 4.06 |
| Melody novelty | Precuneus | R | 5725 | 6, -62, 36 | 5.91 |
|  | Middle temporal gyrus | R | 115 | 64, -20, -14 | 5.75 |
|  | Superior frontal gyrus | R | 688 | 26, 34, 48 | 5.19 |
|  | Superior frontal gyrus | R | 57 | 28, 8, 68 | 5.11 |
|  | Parahippocampal gyrus | L | 67 | -30, -42, -10 | 5.04 |
|  | Middle temporal gyrus | L | 101 | -60, -26, -16 | 4.91 |
|  | Caudate nucleus | L | 82 | -8, 16, 0 | 4.83 |
|  | Superior medial gyrus | R | 57 | 6, 36, 60 | 4.83 |
|  | Middle occipital gyrus | L | 85 | -48, -78, 4 | 4.75 |
|  | Inferior occipital gyrus | L | 53 | -50, -64, -12 | 4.67 |
|  | Cerebellum | L | 101 | -40, -70, -46 | 4.61 |
|  | Middle frontal gyrus | R | 58 | 28, 56, 2 | 4.34 |
|  | Medial prefrontal cortex | L | 114 | -14, 44, -2 | 4.13 |
| Instrument familiarity | Supplementary motor area | R | 19 | 2, 2, 58 | 4.21 |
|  | Premotor cortex | R | 12 | 2, -6, 64 | 4.01 |
|  | Precentral gyrus | L | 11 | -22, -22, 74 | 3.87 |
|  | Middle temporal gyrus | L | 14 | -62, -32, -4 | 3.83 |
|  | Superior occipital gyrus | L | 12 | -16, -84, 32 | 3.56 |
| Dissonance | Inferior parietal lobule | L | 12 | -48, -32, 42 | 3.74 |

Activations are shown for the pooled effect across participant groups (conducted using t-contrasts) in each first-level contrast, significant at an uncorrected threshold of *P* < 0.001 over the whole brain. Peak coordinates are in Montreal Neurological Institute standard space. Only clusters greater than a certain size are shown, due to the large number of activations observed: for timbre change, melody familiarity, melody novelty, k > 50; for melody constancy, instrument familiarity, dissonance, k > 10.

**Table S6. Interaction effect of group with each principal contrast assessing musical object processing**

| **Analysis** | **Region** | **Side** | **Cluster size**  (voxels) | **Peak coordinates** (mm) | **Z-score** |
| --- | --- | --- | --- | --- | --- |
| Timbre change | Superior temporal gyrus | R | 29 | 66, -16, 6 | 4.64 |
|  | Middle temporal gyrus | R | 43 | 64, -38, 4 | 4.59 |
|  | Middle temporal gyrus | L | 32 | -66, -40, 6 | 4.30 |
| Melody familiarity | Supplementary motor area | L | 437 | -6, 0, 62 | 5.79 |
|  | Precuneus | R | 1159 | 6, -62, 36 | 5.52 |
|  | Temporal pole | L | 638 | -52, 12, -4 | 5.20 |
|  | Precentral gyrus | L | 148 | -52, -6, 48 | 5.16 |
|  | Middle temporal gyrus | R | 74 | 44, -72, 12 | 5.02 |
|  | Middle occipital gyrus | L | 202 | -42, -76, 36 | 4.94 |
|  | Superior temporal gyrus | L | 115 | -50, -40, 18 | 4.87 |
|  | Angular gyrus | R | 300 | 52, -60, 32 | 4.85 |
|  | Middle frontal gyrus | R | 79 | 40, 22, 42 | 4.52 |
|  | Superior frontal gyrus | R | 65 | 26, 34, 48 | 4.38 |
|  | Inferior parietal lobule | L | 87 | -50, -52, 42 | 3.96 |
| Melody constancy | Supplementary motor area | R | 4 | 14, 24, 64 | 3.51 |
|  | Postcentral gyrus | R | 5 | 12, -28, 60 | 3.51 |
| Melody novelty | Supplementary motor area | L | 452 | -6, 0, 62 | 5.90 |
|  | Precuneus | R | 1274 | 6, -62, 36 | 5.61 |
|  | Insula | R | 67 | 48, 6, -4 | 5.35 |
|  | Temporal pole | L | 684 | -52, 12, -4 | 5.27 |
|  | Precentral gyrus | L | 146 | -52, -6, 48 | 5.15 |
|  | Middle temporal gyrus | R | 82 | 44, -72, 12 | 5.09 |
|  | Middle occipital gyrus | L | 223 | -42, -76, 36 | 4.98 |
|  | Supramarginal gyrus | L | 134 | -50, -40, 32 | 4.87 |
|  | Angular gyrus | R | 312 | 52, -60, 32 | 4.87 |
|  | Middle frontal gyrus | R | 78 | 40, 22, 42 | 4.47 |
|  | Superior frontal gyrus | R | 66 | 26, 34, 48 | 4.45 |
|  | Inferior parietal lobule | L | 91 | -48, -54, 48 | 3.99 |
| Instrument familiarity | Heschl’s gyrus | L | 148 | -36, -24, 6 | 5.19 |
|  | Heschl’s gyrus | R | 186 | 40, -24, 12 | 5.09 |
| Dissonance | Middle temporal gyrus | R | 15 | 62, -24, -10 | 4.08 |

Activations are shown for the interaction of participant group (conducted using F-contrasts) with each first-level contrast significant at an uncorrected threshold of *P* < 0.001 over the whole brain. Coordinates are in Montreal Neurological Institute standard space. Only clusters greater than a certain size are shown due to a large number of activations observed: for timbre change, k > 25; for melody familiarity, melody novelty and instrument familiarity, k > 50; for melody constancy, k > 3; for dissonance, k > 10.

**Table S7. Functional activations for auditory stimulation in each diagnostic group**

| **Group** | **Region** | **Side** | **Cluster size**  (voxels) | **Peak coordinates**  (mm) | **Z-score** | **P_FWE_-value** |
| --- | --- | --- | --- | --- | --- | --- |
| HV | Heschl’s gyrus | L | 291 | -36, -26, 8 | 7.50 | < 0.001 |
|  |  | R | 264 | 40, -20, 4 | 7.01 | < 0.001 |
| AD | Heschl’s gyrus | R | 200 | 40, -20, 4 | 6.40 | < 0.001 |
|  |  | L | 264 | -44, -20, 2 | 5.96 | < 0.001 |
| tvFTD | Heschl’s gyrus | R | 184 | 52, -8, 0 | 5.88 | < 0.001 |
|  |  | L | 156 | -48, -14, 0 | 5.66 | < 0.001 |

Peak activations are significant (*P* < 0.05) after family-wise error correction within the pre-specified anatomical region of interest, here Heschl’s gyrus (see also Figure S4). Peak coordinates are in Montreal Neurological Institute standard space. AD, patients with Alzheimer’s disease; HV, healthy volunteers; L, left; R, right; tvFTD, patients with temporal variant frontotemporal dementia.

**Table S8. Post-scan music task performance for each participant group**

| Test | HV | AD | tvFTD |  |
| --- | --- | --- | --- | --- |
|  | n = 25 | n = 20 | n = 15 |  |
| Timbre change^a^ |  |  |  |  |
| Sensitivity | 0.99 (0.02) | 0.91 (0.10) | 0.99 (0.04) |  |
| Hit rate | 0.96 (0.07) | 0.78 (0.16) | 0.97 (0.10) |  |
| False alarm rate | 0.01 (0.02) | 0.07 (0.11) | 0.01 (0.03) |  |
| Melody familiarity |  |  |  |  |
| Sensitivity | 0.99 (0.01) | 0.95 (0.05) | 0.97 (0.04) |  |
| Hit rate | 0.98 (0.03) | 0.90 (0.10) | 0.94 (0.07) |  |
| False alarm rate | 0.01 (0.03) | 0.07 (0.07) | 0.05 (0.06) |  |
| Melody constancy^b^ |  |  |  |  |
| Sensitivity | 0.99 (0.01) | 0.95 (0.06) | 0.97 (0.03) |  |
| Hit rate | 0.99 (0.03) | 0.90 (0.10) | 0.94 (0.07) |  |
| False alarm rate | 0.01 (0.03) | 0.07 (0.07) | 0.03 (0.06) |  |
| Instrument familiarity^c^ | |  |  |  |
| Sensitivity | | 0.98 (0.04) | 0.89 (0.14) | 0.92 (0.12) |
| Hit rate | | 0.95 (0.08) | 0.81 (0.20) | 0.85 (0.17) |
| False alarm rate | | 0.02 (0.06) | 0.12 (0.14) | 0.07 (0.12) |
| Dissonance detection^d^ |  |  |  |  |
| Sensitivity | 0.92 (0.08) | 0.87 (0.09) | 0.92 (0.08) |  |
| Hit rate | 0.81 (0.15) | 0.69 (0.17) | 0.79 (0.17) |  |
| False alarm rate | 0.07 (0.09) | 0.09 (0.09) | 0.06 (0.07) |  |

Mean (standard deviation) values are provided for all performance metrics for each task, which have a scale from 0 to 1. Sensitivity was calculated using the non-parametric measure, A-prime, in an adapted Excel workbook.^8^ All tests comprised 16 trials, except for the melody familiarity and melody constancy tests which comprised 68 trials. Task descriptions and results of significant group differences in sensitivity are described in the main text; see also Figure 5. Superscripts code numbers of individuals with missing data for that test, as follows: ^a^2 tvFTD; ^b^2 HV, 4 AD, 3 tvFTD; ^c^1 tvFTD; ^d^1 AD, 2 tvFTD. AD, patient group with Alzheimer’s disease; HV, healthy volunteer group; tvFTD, patient group with temporal variant frontotemporal dementia.

**Figure S1. Structural MRI scans of individual tvFTD participants**


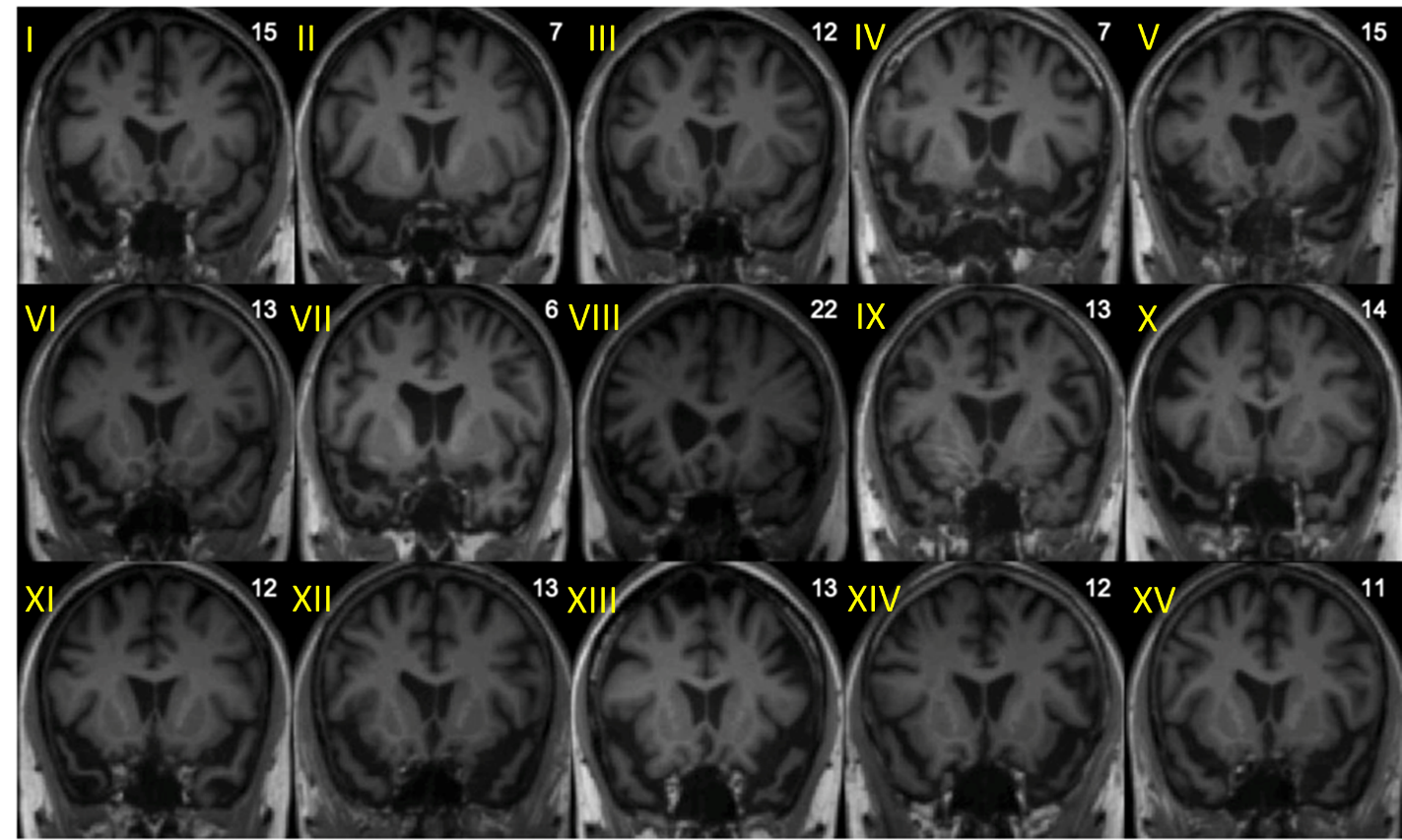


A representative coronal section from each tvFTD participant’s structural T1-weighted structural MRI scan is presented. The scans have been pre-processed according to the procedures described above; coordinates for each section in Montreal Neurological Institute space are indicated in the upper right-hand corner, and the right cerebral hemisphere is shown on the right of each coronal section. Participants I-XI had a syndromic diagnosis of svPPA with predominate left anterior temporal lobe atrophy, whereas participants XII-XV had a syndromic diagnosis of bvFTD with predominate right anterior lobe atrophy. bvFTD, behavioural variant frontotemporal dementia; svPPA, semantic variant primary progressive aphasia; tvFTD, temporal variant frontotemporal dementia.

**Figure S2. Pre-specified neuroanatomical regions of interest**


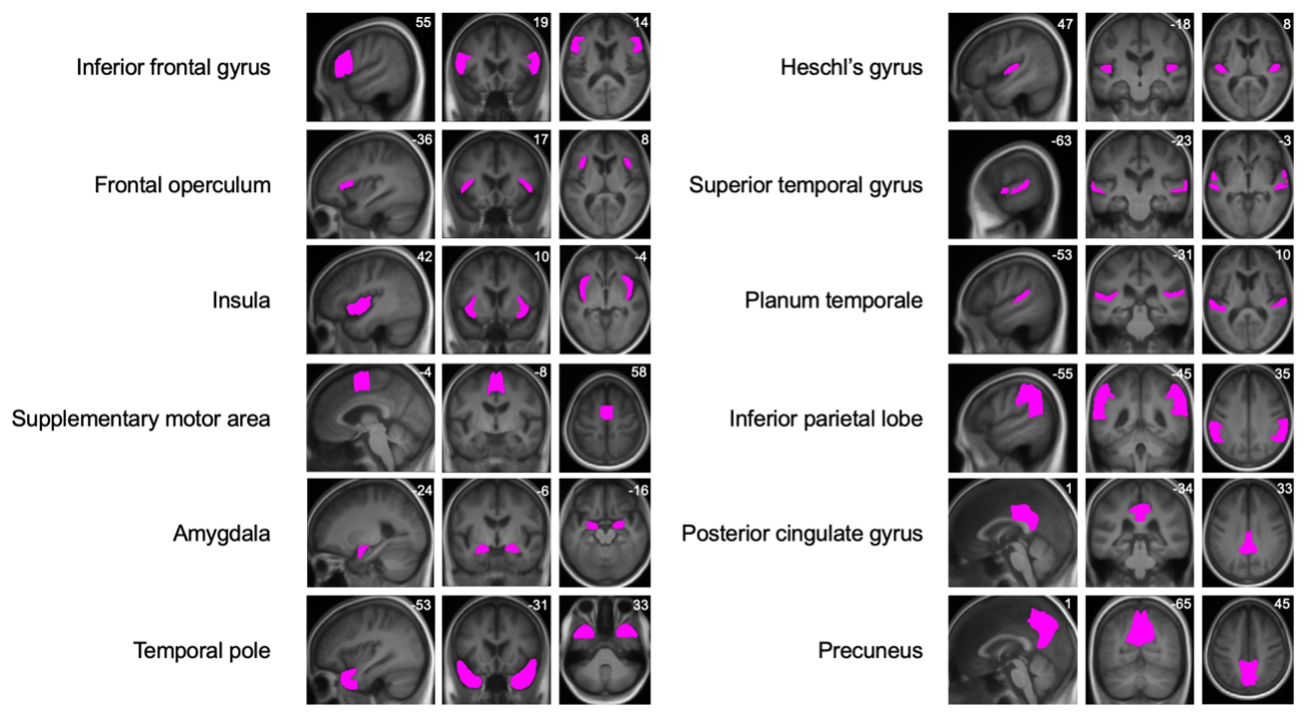


Regions of interest used for small volume correction in the second-level functional MRI analyses (based on pre-specified hypotheses) are shown in magenta, overlaid on sagittal (left), coronal (middle) and axial (right) sections of the group mean structural brain image. The coordinates of each section in Montreal Neurological Institute space are shown on each panel. Regions were derived from the Harvard-Oxford atlas^9^ in FSLView.^10^ Some brain areas were the conjunction of smaller regions. Following the atlas’ naming system: inferior frontal gyrus included pars opercularis and pars triangularis; inferior parietal lobe included angular gyrus, anterior and posterior supramarginal gyrus; superior temporal gyrus included anterior and posterior superior temporal gyrus. We overlaid regions of interest on the group’s mean structural image and performed manual editing to ensure regions only encompassed grey matter volume.

**Figure S3. Grey matter atrophy profiles in each patient group**


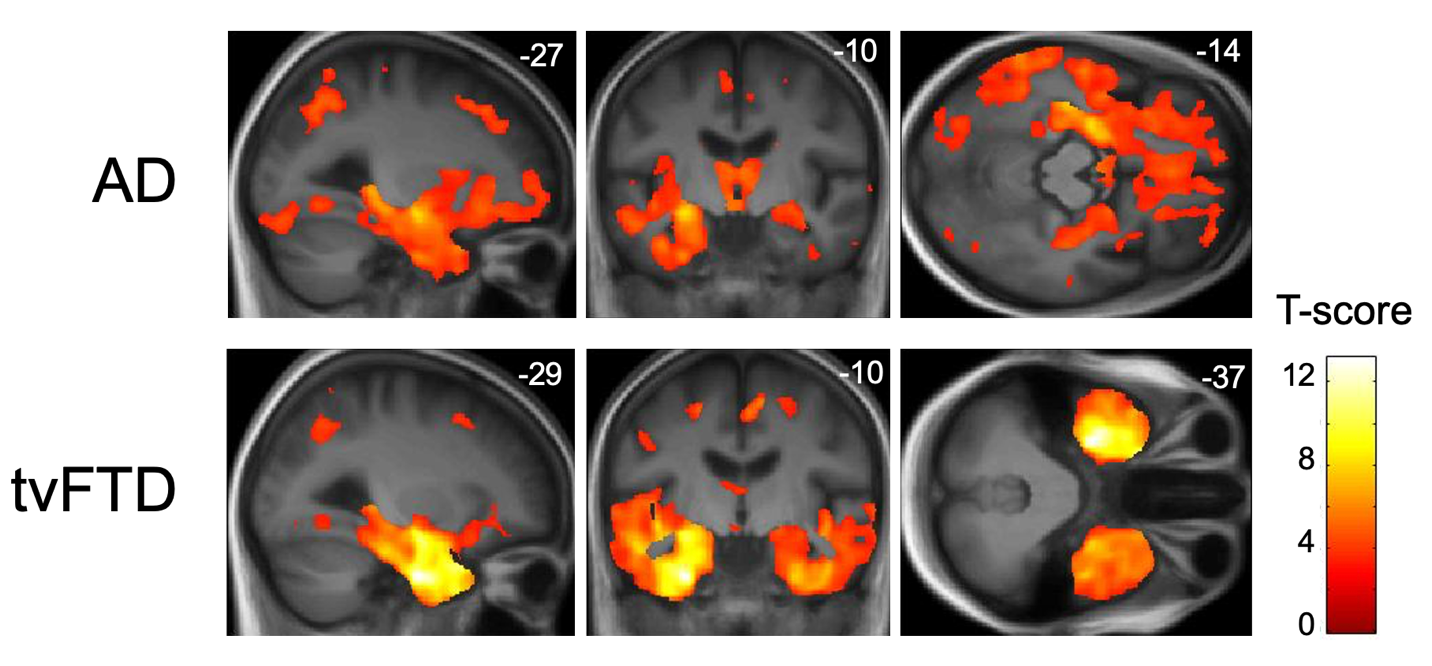


The figure depicts results of the voxel-based morphometry analysis showing statistical parametrical maps of significant regional grey matter atrophy in each patient group compared to HV group, assessed using two-sample t-tests (see also Table S4). *n* = 25, 20, 15 for the HV, AD, tvFTD groups, respectively. Maps are overlaid on sagittal (left), coronal (middle), and axial (right) sections of the group mean structural brain image and thresholded at an uncorrected significance level of *P* < 0.001 for display purposes; local maxima are all significant at *P* < 0.05 after family-wise error correction for multiple voxel-wise comparisons over the whole brain. The coordinates for each section in Montreal Neurological Institute space are indicated; the right cerebral hemisphere is displayed on the right of each coronal section and the bottom of each axial section. The colour bar codes voxel-wise T-scores for between-group grey matter differences. AD, patient group with Alzheimer’s disease; HV, healthy volunteer group; tvFTD, patient group with temporal variant frontotemporal dementia.

**Figure S4. Brain activation profile associated with auditory stimulation**


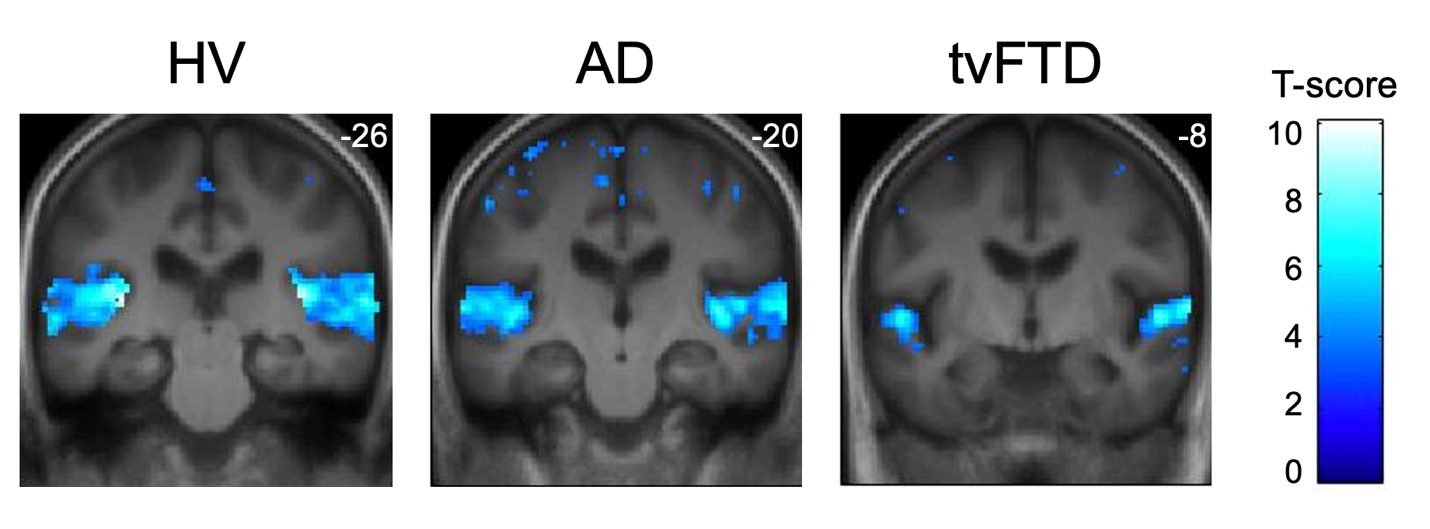


The figure shows statistical parametric maps of significant brain activation in the group-level functional MRI analysis for the t-contrast of all music conditions versus the silence baseline in each participant group (see also Table S7). *n* = 25, 20, 15 for the HV, AD, tvFTD groups, respectively. Activations are overlaid on coronal sections of the group mean structural brain image and thresholded at an uncorrected significance level of *P* < 0.001 for display purposes; local maxima are all significant at *P* < 0.05 after family-wise error correction for multiple voxel-wise comparisons within the pre-specified anatomical region of interest, here Heschl’s gyrus bilaterally. The coordinates of each section in Montreal Neurological Institute space are indicated; the right cerebral hemisphere is displayed on the right in all sections. The colour bar (right) codes voxel-wise T-scores. AD, patient group with Alzheimer’s disease; HV, healthy volunteer group; tvFTD, patient group with temporal variant frontotemporal dementia.

**Figure S5 Brain activation for each principal contrast assessing musical object processing pooled across all participant groups**


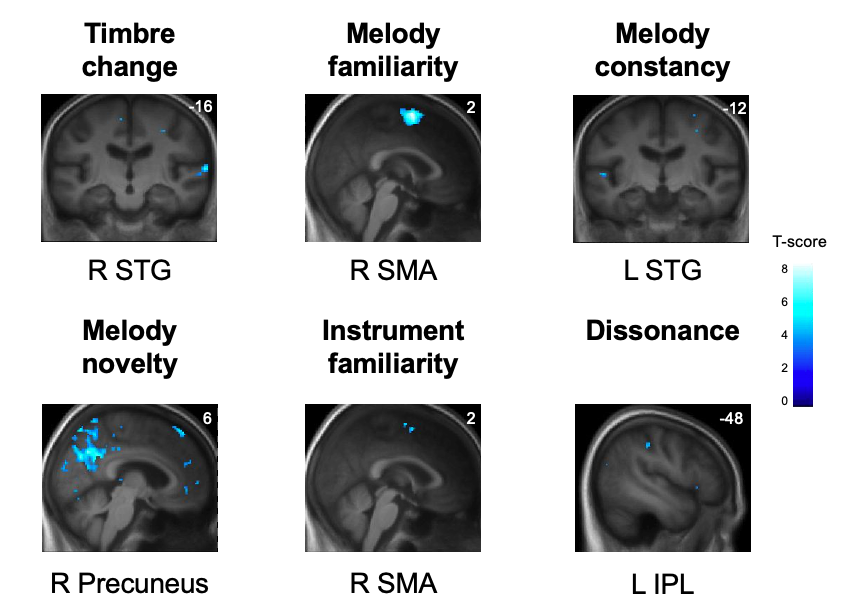


The figure shows statistical parametric maps of brain activation in the group-level fMRI analysis for each principal contrast assessing musical object processing pooled across all participant groups (n = 60); see also Table S5. T-contrasts were used to assess the pooled activation. Activations are overlaid on coronal or sagittal sections of the group mean structural brain image and thresholded at an uncorrected level of *P* < 0.001. The associated coordinate in Montreal Neurological Institute space is indicated in the upper right-hand corner of each brain image; the left cerebral hemisphere is displayed on the left of each coronal section. The colour bar codes voxel-wise T-scores. IPL, inferior parietal lobule; L, left; R, right; SMA, supplementary motor area; STG, superior temporal gyrus.

**Figure S6 Brain activation for the interaction effect of group with each principal contrast assessing musical object processing**


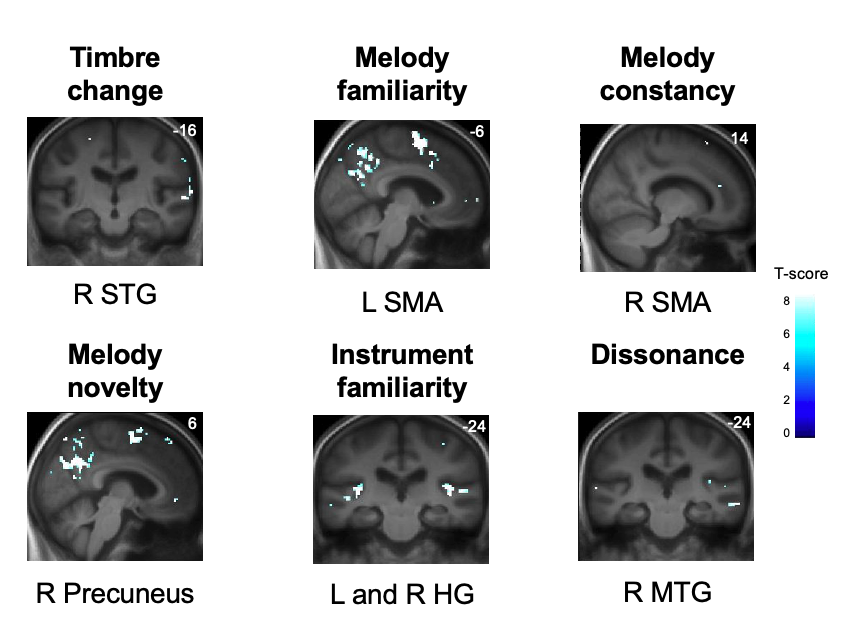


The figure shows statistical parametric maps of brain activation in the group-level fMRI analysis for the interaction effect of group with each principal contrast assessing musical object processing (n = 60); see also Table S6. F-contrasts were used to assess the interaction effect. Activations are overlaid on coronal or sagittal sections of the group mean structural brain image and thresholded at an uncorrected level of *P* < 0.001. The associated coordinate in Montreal Neurological Institute space is indicated in the upper right-hand corner of each brain image; the left cerebral hemisphere is displayed on the left of each coronal section. The colour bar codes voxel-wise T-scores. HG, Hechl’s gyrus; L, left; MTG, middle temporal gyrus; R, right; SMA, supplementary motor area; STG, superior temporal gyrus.

**Supplementary references**

1. Hébert S, Peretz I. Recognition of music in long-term memory: Are melodic and temporal patterns equal partners? *Memory & cognition*. 1997;25(4):518-533.

2. Fisher NJ, Tierney MC, Snow WG, Szalai JP. Odd/Even short forms of the Boston Naming Test: preliminary geriatric norms. *Clin Neuropsychol*. Aug 1999;13(3):359-64. doi:10.1076/clin.13.3.359.1742

3. Calamia M, Markon K, Denburg NL, Tranel D. Developing a short form of Benton's Judgment of Line Orientation Test: an item response theory approach. *Clin Neuropsychol*. May 2011;25(4):670-84. doi:10.1080/13854046.2011.564209

4. Goll JC, Kim LG, Hailstone JC, et al. Auditory object cognition in dementia. *Neuropsychologia*. Jul 2011;49(9):2755-65. doi:10.1016/j.neuropsychologia.2011.06.004

5. Audiology BSo. *Pure-tone air-conduction and bone-conduction threshold audiometry with and without masking*. 2018. https://www.thebsa.org.uk/wp-content/uploads/2023/10/OD104-32-Recommended-Procedure-Pure-Tone-Audiometry-August-2018-FINAL-1.pdf

6. Lin F, Reed N. The Pure-Tone Average as a Universal Metric—Knowing Your Hearing. *JAMA Otolaryngology - Head and Neck Surgery*. 12/23 2020;147doi:10.1001/jamaoto.2020.4862

7. Ridgway GR, Omar R, Ourselin S, Hill DL, Warren JD, Fox NC. Issues with threshold masking in voxel-based morphometry of atrophied brains. *Neuroimage*. Jan 1 2009;44(1):99-111. doi:10.1016/j.neuroimage.2008.08.045

8. *Signal detection theory calculator 1.2*. 2017. https://www.researchgate.net/profile/Justin_Gaetano2/

9. Desikan RS, Segonne F, Fischl B, et al. An automated labeling system for subdividing the human cerebral cortex on MRI scans into gyral based regions of interest. *Neuroimage*. Jul 1 2006;31(3):968-80. doi:10.1016/j.neuroimage.2006.01.021

10. Jenkinson M, Beckmann CF, Behrens TE, Woolrich MW, Smith SM. Fsl. *Neuroimage*. Aug 15 2012;62(2):782-90. doi:10.1016/j.neuroimage.2011.09.015
